# Effects of Habitat and Fruit Scent on the Interactions between Short-tailed Fruit Bats and *Piper* Plants

**DOI:** 10.1093/iob/obae028

**Published:** 2024-07-29

**Authors:** S Sil, F Visconti, G Chaverri, S E Santana

**Affiliations:** Department of Chemistry, University of Washington, Seattle, WA 98195, USA; Department of Biology, University of Washington, Seattle, WA 98195, USA; Burke Museum of Natural History and Culture, University of Washington, Seattle, WA 98195, USA; Sede del Sur, Universidad de Costa Rica, Golfito 60701, Costa Rica; Smithsonian Tropical Research Institute, 0843-03092 Balboa, Ancón, Panamá; Department of Biology, University of Washington, Seattle, WA 98195, USA; Burke Museum of Natural History and Culture, University of Washington, Seattle, WA 98195, USA

## Abstract

*Piper* is a mega-diverse genus of pioneer plants that contributes to the maintenance and regeneration of tropical forests. In the Neotropics, *Carollia* bats use olfaction to forage for *Piper* fruit and are a main disperser of *Piper* seeds via consumption and subsequent defecation during flight. In return, *Piper* fruits provide essential nutrients for *Carollia* year-round. There is evidence that the types and diversity of *Piper* frugivores are influenced by the primary habitat type of different *Piper* species (forest and gap), with forest *Piper* depending more on bats for seed dispersal; however, this pattern has not been tested broadly. We aimed to characterize and compare the interactions between *Carollia* and *Piper* across forested and gap habitats, and further investigate whether differences in fruit traits relevant to bat foraging (i.e., scent) could underlie differences in *Carollia-Piper* interactions. We collected nightly acoustic ultrasonic recordings and 24 h camera trap data in La Selva, Costa Rica across 12 species of *Piper* (six forest, six gap) and integrated this information with data on *Carollia* diet and *Piper* fruit scent. Merging biomonitoring modalities allowed us to characterize ecological interactions in a hierarchical manner: from general activity and presence of bats, to visitations and inspections of plants, to acquisition and consumption of fruits. We found significant differences in *Carollia-Piper* interactions between forested and gap habitats; however, the type of biomonitoring modality (camera trap, acoustics, diet) influenced our ability to detect these differences. Forest *Piper* were exclusively visited by bats, whereas gap *Piper* had a more diverse suite of frugivores; the annual diet of *Carollia*, however, is dominated by gap *Piper* since these plants produce fruit year-round. We found evidence that fruit scent composition significantly differs between forest and gap *Piper*, which highlights the possibility that bats could be using chemical cues to differentially forage for gap vs. forest *Piper*. By integrating studies of *Piper* fruit scent, plant visitation patterns, and *Carollia* diet composition, we paint a clearer picture of the ecological interactions between *Piper* and *Carollia*, and plant-animal mutualisms more generally.

## Resumen (Spanish)

*Piper* es un género megadiverso de plantas pioneras que contribuyen al mantenimiento y regeneración de los bosques tropicales. En el Neotrópico, los murciélagos de género *Carollia* utilizan el sentido del olfato para encontrar frutos de *Piper* y son los principales dispersores de estas plantas a través del consumo de los frutos y posterior defecación de las semillas durante el vuelo. A cambio, los frutos de *Piper* proporcionan nutrientes esenciales para *Carollia* durante todo el año. Existe evidencia de que los tipos y la diversidad de frugívoros que consumen *Piper* están influenciados por el tipo de hábitat primario (bosques y claros) de las diferentes especies, y que los *Piper* de bosque dependen más de los murciélagos para la dispersión de semillas. Sin embargo, este patrón no ha sido corroborado ampliamente. Nuestro objetivo fue caracterizar y comparar las interacciones entre *Carollia* y *Piper* en hábitats boscosos y en claros, e investigar más a fondo si las diferencias en los rasgos de las frutas relevantes para la búsqueda de frutos por los murciélagos (es decir, el olor) podrían explicar las diferencias en las interacciones entre *Carollia* y *Piper*. Para ello, compilamos grabaciones acústicas ultrasónicas durante la noche y videos con cámaras trampa por 24 horas/día en La Selva, Costa Rica, para 12 especies de *Piper* (6 de bosque, 6 de claros) e integramos esa información con datos sobre la dieta de *Carollia* y el aroma de los frutos de *Piper*. Esta combinación de modalidades de biomonitoreo nos permitió caracterizar las interacciones ecológicas de manera jerárquica: desde la actividad general y la presencia de murciélagos, sus visitas e inspecciones de las plantas, hasta la adquisición y consumo de frutos. Encontramos diferencias significativas entre hábitats boscosos y claros en las interacciones de *Carollia* y *Piper*;

sin embargo, el tipo de modalidad de biomonitoreo (cámara trampa, acústica, dieta) influyó en nuestra capacidad de detectar estas diferencias. Los *Piper* de bosque fueron visitados exclusivamente por murciélagos, mientras que los *Piper* de claros tuvieron un grupo más diverso de frugívoros. La dieta anual de *Carollia*, sin embargo, está dominada por *Piper* de claros, ya que estas plantas producen frutos durante todo el año. Encontramos evidencia de que la composición del aroma de los frutos difiere significativamente entre los *Piper* de bosque y de claros, lo que resalta la posibilidad que los murciélagos puedan estar usando señales químicas para forrajear diferencialmente por *Piper* de estas dos categorías. La integración de estudios del aroma de los frutos de *Piper*, patrones de visita a las plantas y composición de la dieta de *Carollia* nos permitió generar una imagen más clara sobre las interacciones ecológicas entre *Piper* y *Carollia* y de los mutualismos planta-animal en general.

## Introduction

The interactions between plants and animals are crucial both for the ecology and evolution of species and are responsible for maintaining and rebuilding healthy ecosystems (Whelan et al. 2008; Kunz et al. 2011). Bats, the only flying mammals, are particularly important in tropical and subtropical regions for the pollination and seed dispersal of hundreds of plant species, forming intricate networks mediated by morphological and behavioral co-adaptations (Mello et al. 2019). In the Neotropics, the mutualism between two highly abundant and widespread taxa–short-tailed fruit bats (*Carollia* spp.; nine species) and *Piper* plants (*Piper* spp.; ∼1200 Neotropical species)–is an example of such a relationship. Via consumption of infructescences (from here on referred to as fruits) and subsequent defecation of seeds, *Carollia* disperse early, mid, and late succession *Piper* species, henceforth mitigating the changes to populations and community structure caused by deforestation and other forms of habitat alteration in tropical environments (Jones et al. 2009). In turn, *Piper* fruits make up to 50–80% of *Carollia*’s annual diet (Lopez and Vaughan 2007; Maynard et al. 2019; Santana et al. 2021) and provide a consistent source of nutrients for these bats (Fleming 1991; Gelambi and Whitehead 2023).

While the *Carollia-Piper* mutualism has been characterized on many fronts, the patterns of interactions between these bats and plants across habitats have received less attention. This information is critical for understanding how dynamic these interactions are across space, the role of these species in local ecological communities, and their importance in ecosystem resilience. At one Panamanian site, Thies and Kalko (2004) found that *Piper* species differed in their time of ripening and seed disperser spectrum, and thereby provided the broad characterization of two major *Piper* ecotypes: “forest” *Piper*, which exhibit short and staggered fruiting peaks, fruits that ripen in the evening, and a narrow spectrum of frugivores (bats; *C. castanea, C. perspicillata*), and “gap” *Piper* with extended fruiting seasons, fruits that ripen early in the morning, and a larger range of seed dispersers (bats, birds, insects) (Thies and Kalko 2004). To an extent, this classification also describes the habitat and location of *Piper* plants; forest *Piper* grow in the understory and gap *Piper* grow in open habitats. However, forest and gap *Piper* can also be located in relatively close proximity to each other –for example, when gap *Piper* grows in forest clearings and trails– and, because *Piper* are found across most successional stages, these plants can also be classified into finer habitat categories: early-succession (gap), mid-succession, and late-succession (forest). Many mid-succession *Piper* species cannot be neatly categorized into a forest or gap ecotype as they fall somewhere in between (S.E.S. pers. obs.).

Abiotic factors have been posited to be the primary drivers of differences in flowering phenology between forest and gap *Piper*, whereas the spectra of seed dispersers in each habitat is thought to drive differences in fruiting patterns (staggered vs. continuous; morning vs. evening ripening) (Thies and Kalko 2004). That is, the long and overlapping fruiting periods of gap *Piper* species could be associated with a larger spectrum of dispersers that would mitigate the challenges of seed dispersal into spatially unpredictable germination sites (Thies and Kalko 2004). While evidence points this might be true for the one site studied thus far, it is not known whether differences in frugivore spectra between forest and gap *Piper* ecotypes are generalizable to other *Piper* species and sites in the Neotropics. As a first goal of this study, we aim to help fill this knowledge gap by contrasting frugivore-*Piper* interaction patterns across *Piper* species and habitats in Costa Rica, which we documented via three biomonitoring modalities: ultrasonic acoustic recordings, camera trap videos, and dietary analyses. We hypothesize that ecotype (forest, gap) and habitat (early-, mid-, late-succession) play a role in defining the community of frugivores that feed from *Piper* plants, and predict there will be a greater diversity of frugivores visiting gap (early-succession) *Piper* compared to forest (mid/late-succession) *Piper* species, with the latter being consumed exclusively by bats (consistent with the Thies and Kalko 2004 study).

Frugivorous Neotropical bats (including *Piper* specialist *Carollia*) integrate across sensory modalities to locate and acquire ripe fruit; they use vision to detect fruit color, olfaction to detect fruit scent volatiles, and echolocation to collect information on the location and shape of fruits (Kalko and Condon 1998; Von Helversen and Von Helversen 1999; Schwab and Pettigrew 2005; Hodgkison et al. 2013; Leiser-Miller et al. 2020; Santana et al. 2021). Behavioral experiments have further shown *Carollia* primarily utilizes olfaction to locate fruiting patches and then echolocation when approaching their target before snagging fruit, and these bats only seem to attempt consumption of *Piper* fruits when appropriate scent cues are present (Thies et al. 1998; Leiser-Miller et al. 2020). Therefore, our second goal was to investigate the role of fruit traits as possible mediators of the differences in frugivore visitation patterns between *Piper* ecotypes, with a focus on traits known to be relevant to bat foraging behavior. Most Neotropical *Piper* plants produce green fruits with small seeds and a distinctive bouquet of volatile organic compounds (VOCs) when ripe (Thies and Kalko 2004; Santana et al. 2021). These VOCs are secondary metabolites that can act as signals adapted to target mutualistic frugivores, and include terpenes, alcohols, and carbonyl compounds (Santana et al. 2021). Previous studies have also shown that *Piper*-specialist *Carollia* mainly rely on olfactory cues for selecting ripe *Piper* fruits and prefer samples enriched with the *Piper* VOCs 2-heptanol and alpha-caryophyllene, indicating that these compounds could have a role in attracting bats to ripe *Piper* fruits (Thies et al. 1998; Leiser-Miller et al. 2020; Santana et al. 2021). An aspect that remains unknown, however, is the extent to which fruit ripeness and the strength of the chemical signal generated by its scent may influence bat foraging behavior, including how frequently bats visit different *Piper* species. For example, *Piper* species with strong scent signals or VOCs preferred by bats might experience higher visitation and consumption rates than plants without these signals or VOCs. Using previously published fruit scent chemical data, we test the hypothesis that differences in fruit scent VOCs between forest and gap *Piper* contribute to differences in how attractive they are to bats, and hence influence bat visitation and consumption patterns across ecotypes.

Altogether, we applied an integrative approach for a detailed comparison of the *Carollia-Piper* mutualism across habitats, and investigated the resulting trends in the context of *Piper* fruit chemical signals relevant to bat consumption patterns. Working in a Costa Rican site, we evaluated the visitation frequency of bats and other frugivores to *Piper* plants via nightly ultrasonic acoustic recordings and camera traps and complemented these data with our published data on *Piper* consumption by *Carollia* and *Piper* fruit VOCs, all collected at the same site. We find that this approach allows us to characterize ecological interactions in a hierarchical manner: from general activity and presence of bats, to visitations and inspections of plants, to acquisition and consumption of fruits, to the molecules potentially mediating these interactions. By describing significant differences in *Carollia-Piper* interactions and fruit scent composition between forest and gap *Piper*, our study thereby provides novel insights on the *Carollia*-*Piper* mutualism and a foundation for future experimental work within this important ecological system.

## Methods

### Study site

**Fig. 1:**
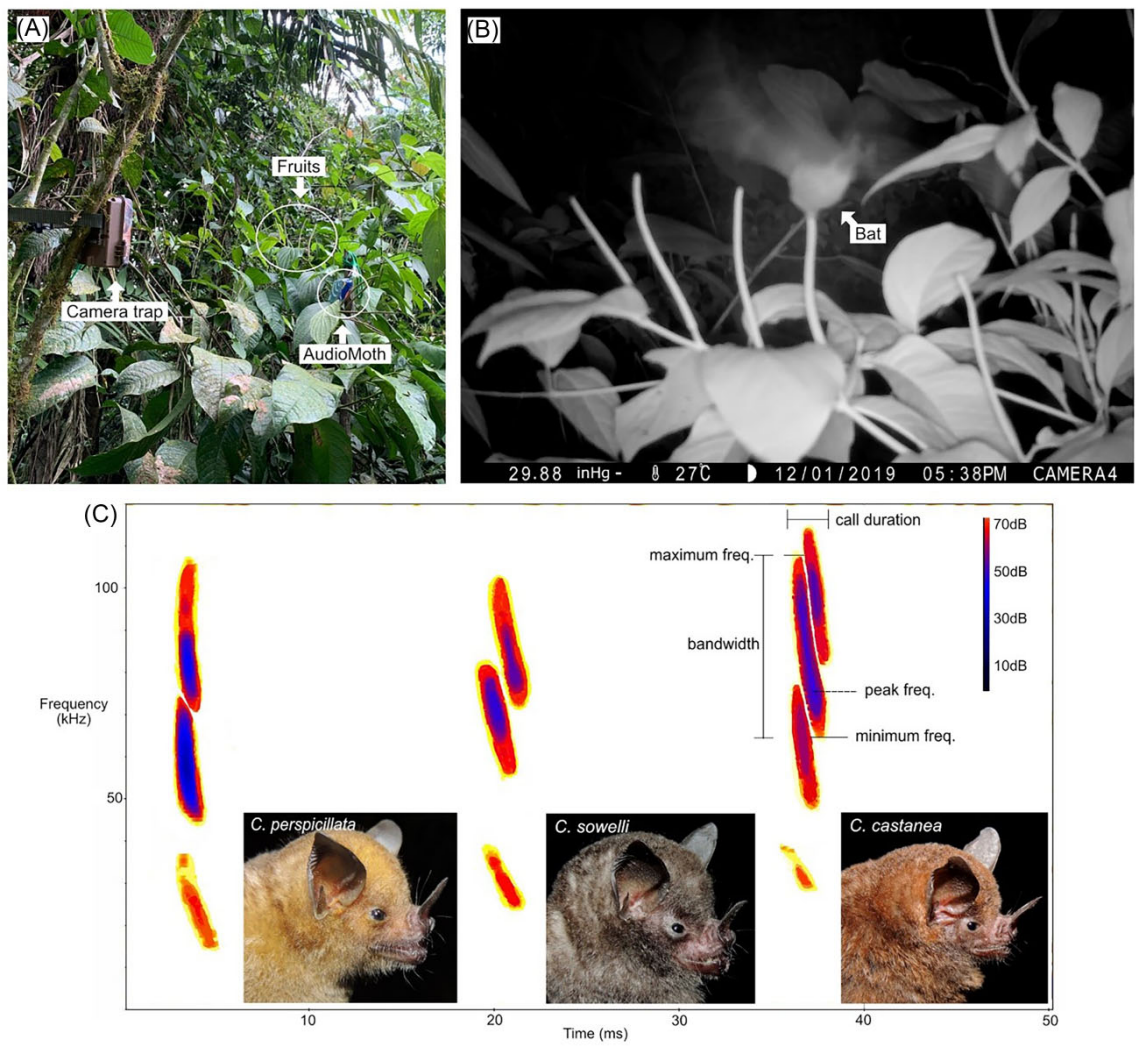
Experimental setup including camera trap and ultrasonic acoustic recorder (AudioMoth) deployed at a *Piper sancti-felicis* plant in the field (A), a video frame showing a bat collecting a fruit at the same plant (B; video available as a supplementary file [Supplementary Video 1]), and reference echolocation calls for *Carollia perspicillata, C. sowelli*, and *C. castanea* (C; spectrograms generated in BatSound v4.4). Analysis of acoustic data was performed using the parameters marked in the spectrogram (call duration, peak frequency, minimum frequency, maximum frequency, and bandwidth of the main harmonic; see Table S2). *Carollia* photos credit: David Villalobos Chaves.

The study was conducted at the Organization for Tropical Studies’ La Selva Biological Reserve, Costa Rica (herein La Selva). The reserve comprises 1600 ha of protected area spanning primary premontane and tropical wet forest, secondary forest, and abandoned agricultural land. *Piper* is highly diverse at La Selva, with over 50 recognized species (OTS 2023), which can be roughly classified into the gap (early-succession) or forest (mid- to late-succession) ecotypes of Thies and Kalko (2004) (see Table 1 and Supplementary Information Table S1 for ecotype and habitat classifications; Greig 1993). Three *Carollia* species (Chiroptera: Phyllostomidae) occur at La Selva (*C. castanea*, 11 g; *C. sowelli*, 18 g; and *C. perspicillata*, 21 g; Santana et al. 2021; Fig. 1c); these are some of the most abundant bats at the site year-round and coexist with about 62 other bat species (OTS 2023). This research was conducted under Costa Rican permit SINAC-ACC-PI-R-107-2019. All procedures were approved by the Institutional Animal Care and Use Committee of the University of Washington, Seattle, USA (protocol #4307-02).

**Table 1:** The 12 *Piper* species at La Selva, Costa Rica, focal to this study, their habitat classification, number of fruit collections and visitation events by bats and other frugivores recorded by camera traps, and the average % of each species in the annual diet of *Carollia sowelli, C. perspicillata*, and *C. castanea* (from the literature, see text for sources).

*Piper* species	Habitat classifications		Fruit collections by bats (camera)	Bat visitations (camera + acoustic)	Other visitations, type of visitor and behavior (^1^: fruit inspection; ^2^:fruit consumption; ^3^: whole plant consumption)	Average % of *Carollia* diet
*P. auritum*	Gap	Early-succession	0	0	–	9.69%
*P. colonense*	Gap	Mid-succession	1	0	9 (hummingbird, Passerini's tanager^2^, wasps^2^, ants^1,2^)	5.62%
*P. multiplinervium*	Gap	Early-succession	0	0	4 (Passerini's tanager^2^, crested guan, golden hooded tanager)	14.66%
*P. reticulatum*	Gap	Mid-succession	3	17	–	5.13%
*P. sancti-felicis*	Gap	Early-succession	1	7	10 (Passerini's tanager^2^, gray four-eyed opossum^1^)	29.15%
*P.* species D	Gap	Mid-succession	0	91	27 (Passerini's tanager^2^)	3.60%
*P. umbricola*	Gap	Early-succession	0	0	–	8.51%
*P. cyanophyllum*	Forest	Mid-succession	1	0	–	0.07%
*P. generalense*	Forest	Mid-succession	6	4	2 (mouse^1^)	1.90%
*P. nudifolium*	Forest	Mid-succession	1	2	2 (hummingbird, tapir^3^)	0.07%
*P. paulowniifolium*	Forest	Mid-succession	0	8	–	1.12%
*P. sublineatum*	Forest	Mid-succession	1	1	–	0.17%

### Recording setup

**Fig. 2:**
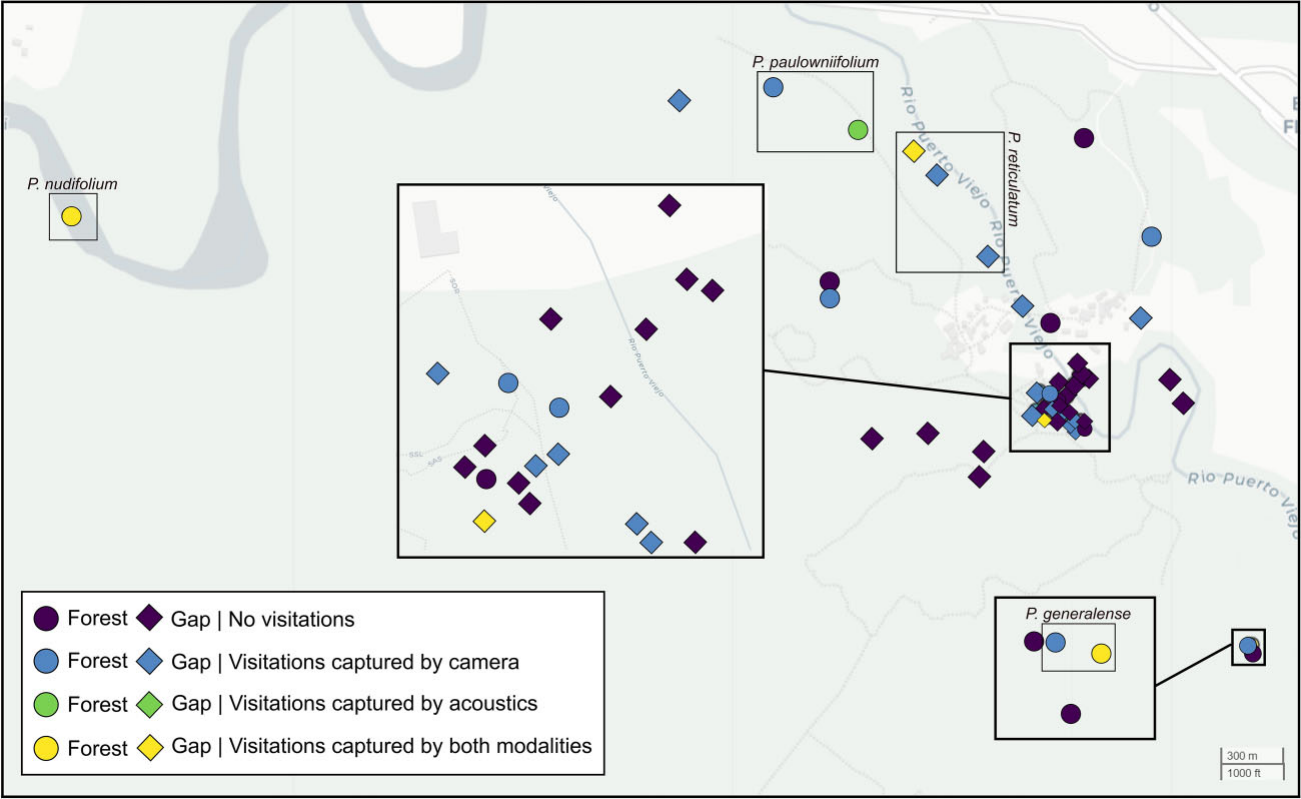
Map of the study area at La Selva Biological Reserve, Costa Rica, showing the locations of all *Piper* plants, within forest and gap habitats, where camera traps and acoustic recorders were deployed. Each plant is color-coded based on whether *Carollia* visitations occurred and how these visitations were documented: by camera traps, acoustic recorders, or both. Groups of *Piper* species showing activity by both camera traps and acoustic recordings are labeled as well.

**Fig. 3:**
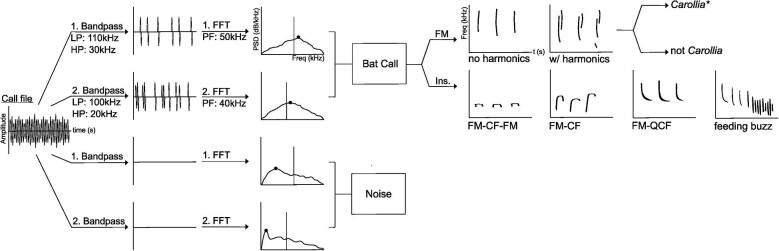
Visual representation of our parallel-processing algorithm developed to first filter (LP: low pass, HP: high pass) through the large acoustic dataset and identify files with bat calls present (PF: peak frequency), and the subsequent criteria used to manually categorize these files into various call types (FM: frequency modulated, Ins: insectivorous). The first run (LP: 110 kHz, HP: 30 kHz) settings were more specific, and the second run (LP: 100 kHz, HP: 20 kHz) settings were more sensitive. FM calls were split into calls with no harmonics and calls displaying harmonics (which were then qualitatively determined to be *Carollia* calls or not). Insectivorous calls were split into FM-CF-FM calls (frequency-modulated, constant-frequency, frequency-modulated), FM-CF calls (frequency-modulated, constant frequency), and FM-QCF (frequency-modulated, quasi-constant frequency) calls. Feeding buzzes were also noted. Results with the total numbers of each call type identified at each *Piper* plant analyzed after filtering can be found in Table S3. *See Fig. 1c for criteria on qualitatively identifying *Carollia* bat calls.

We documented bat activity and behavior at 45 plants across 12 species of *Piper* (six forest, six gap; Table 1) for 1–211 days per plant between 2019 and 2021 (Table S1). We selected *Piper* plants on the basis of three criteria: (1) plants had at least one fully formed (presumed ripe or close-to-ripe) fruit; the fruits of most Neotropical *Piper* species remain a shade of green when ripe but become noticeably plump and softer when they approach ripeness; (2) fruits were accessible to place acoustic recorders and cameras no more than 50 cm (acoustics) or 5 m (cameras) away from fruits (Fig. 1a); (3) plant location maximized spatial distance among plants of the same species (at least 3 m, but typically tens to hundreds of meters apart; Fig. 2, Table S1). For video documentation of frugivores at *Piper* plants, we used motion-activated Browning Advantage Spec Ops Full HD Video Trail Cameras (Browning Trail cameras, Birmingham, AL, USA; Model BTC-8A), which were strapped to trees, lianas, poles, rails, or other available structures and positioned to ensure the fruits were centered within the field of view (Fig. 1a). Cameras were set to capture HD videos at a 1920 × 1080, 60 fps resolution, with motion detection at a minimum of 60 ft. and a trigger speed of 0.4 s. The cameras recorded for 24 h each day, using an infrared function during the night, and set to record for 20 s as soon as movement was detected. Sequential 20-s videos were stored when movement was detected for longer periods of time. For acoustic documentation of bats during the night, we used AudioMoths (Open Acoustic Devices, UK), which are full-spectrum acoustic loggers based on the Gecko processor range from Silicon Labs. We placed these close to fruits (≤50 cm), encased in the AudioMoth IPX7 Waterproof Case. We set AudioMoths to record starting at dusk and to span the known high activity period of *Carollia* (5–8 PM local time), using a sample rate of 256 kHz, medium gain, for 10 s intervals every 20 s. We chose these settings to increase our chances of detecting *Carollia's* relatively “quiet” echolocation calls, and produce a manageable amount of data, respectively. We monitored plants every 1–3 days and stopped video and audio recordings as soon as the focal fruit(s) had been removed from the plant, and no plants were recorded more than once. A few plants, however, were video recorded for a much longer time because we had to leave cameras deployed and unattended during lockdowns and travel restrictions associated with the COVID-19 pandemic.

### Camera trap video analysis

One of us (F.V.) performed video analysis to avoid bias in the results. We analyzed videos collected from camera traps using QuickTime Player 8 on a macOS operating system (Apple Inc., Cupertino, CA, USA), and took note of: the organism(s) observed in the recording to the lowest possible taxonomic level (e.g., bat, tanager, tapir, rodent, and so on), the action performed by the organism (via freestyle identification by F.V.), and the time and date at which this behavior took place. We first observed each 20-s video at normal speed playback to help identify the source of movement, since the camera trap sensor was sometimes triggered by leaves or branches being blown by wind. When an animal was encountered in the videos, we would play the video again at half speed at least once or twice to determine what behavior was being performed. Bats circling plants move at a fast speed; therefore, many videos had to be analyzed two or three additional times at half speed to properly identify behavior. Additionally, we analyzed the videos about 4–5 times at half speed and original speed if they contained activity from more than one animal, such as multiple tanagers, so we could accurately take notes on each individual's behavior. We performed classification of animals that were not bats with the aid of field guides containing physical descriptions and images of the different animal species found across Costa Rica (Garrigues and Dean 2007).

### Acoustic analysis

We compiled a call library of search-phase echolocation call recordings of *C. perspicillata, C. sowelli*, and *C. castanea* to create quantitative and qualitative references for manual *Carollia* echolocation call identification (manual ID; Fig. 1c) in our field data. These calls were recorded with a condenser microphone (microphone capsule CM16, CMPA preamplifier unit, Avisoft Bioacoustics, Berlin, Germany). We generated spectrograms (e.g., Fig. 1c) using RavenPro v. 1.6.2 (512 FFT Hanning window; 95% overlap; K. Lisa Yang Center for Conservation Bioacoustics at the Cornell Lab of Ornithology 2022), and collected the following parameters to act as a quantitative reference: call duration (ms), 90% call duration (ms), minimum frequency (kHz), maximum frequency (kHz), peak frequency (kHz), 95% frequency (kHz), delta frequency (kHz), and 90% bandwidth (kHz) (Table S2), all of which are widely used to characterize echolocation vocalizations (Luo et al. 2019). We found the general frequency ranges and shape of echolocation calls to be relevant as well for qualitative manual ID (below). However, considering the lack of published *Carollia* spp. call library data, we collected extra parameters to increase the reliability of our manual ID method and to serve for future reference (Table S2). This preliminary step of analyzing focal call data and creating call guides is essential for proper acoustic identification, as bat calls may be only accurately identified by known qualitative and/or quantitative measures (Fraser et al. 2020). However, our focal call parameters showed significant overlap between *C. castanea, C. perspicillata*, and *C. sowelli* echolocation calls (Fig. 1c); therefore, we aimed to mainly identify calls to the *Carollia* genus when possible. Generally, identification to the species level is especially difficult for low-duty cycle call species such as *Carollia* spp. because their calls exhibit the most intraspecific and intraindividual flexibility associated with different tasks and habitat effects (Russo et al.^2017^).

The main challenge in analyzing passive acoustic recordings from a tropical forest site is environmental clutter: humidity, vegetation, and foliage, and other animal sounds can cause echoes and additional noise into the path of the incoming sound (Fraser et al. 2020). These factors are unavoidable; as a result, our field data contained significant background noise. Additionally, we accrued a massive dataset which was impractical for one researcher to go through manually (characteristic of most experiments utilizing passive acoustic monitoring [Fraser et al. 2020]); therefore, we used a semi-automated method to sort through our large, noisy acoustic dataset. We developed a filtering program in MATLAB v. 9.12.0 (The MathWorks Inc. 2022; Fig. 3) which sorted through the dataset using a bandpass filter (butterworth) to filter out noise below the minimum frequency threshold of *Carollia* calls (approximately 45kHz, according to our focal parameters; Table S2). Then, the program generated a power spectrum (pwelch) used to filter the acoustic files into two categories: containing bat calls (above a threshold frequency) or mainly consisting of noise (below the threshold frequency). The threshold frequency, like the bandpass filter, was chosen based on the focal call data parameters (in this case, the peak frequencies of three *Carollia* species). If the peak frequency of the filtered signal was in the range of *Carollia* search-phase echolocation call peak frequency (anywhere from 60 to 80 kHz depending on the species [Table S2]), this indicated high activity within that frequency and the likely presence of bats in the habitat where the calls were recorded. The algorithm ran on the dataset twice with two different bandpass and peak frequency threshold parameters; once with more sensitive parameters (type II error) and once with more specific parameters (type I error). The overall goal of this program was to sort through the large dataset and set aside a reasonable number of files for a researcher trained on spectrogram analysis of *Carollia* focal search-phase echolocation calls (S.Sil) to analyze manually. We deemed this hybrid approach the best way to deal with the large dataset and noise present in the data considering that completely automated identification can generate significant error rates which could influence our characterization of *Carollia-Piper* interactions across habitats (Russo and Voigt 2016; Rydell et al. 2017; Barré et al. 2019). Subsequently, one of us (S.Sil) carried out manual identification of bat calls across individual *Piper* plants and species to avoid bias in the results.

As shown in Fig. 3, we classified bat calls based on our quantitative and qualitative references. We generated spectrograms to view calls using RavenPro v. 1.6.2 (Cornell Lab of Ornithology, Ithaca, NY, USA) and BatSound (Pettersson Elektronik, Uppsala, Sweden) v. 4.4 (512 FFT Hanning window, 95% overlap; Pettersson Elektronik AB 2016; K. Lisa Yang Center for Conservation Bioacoustics at the Cornell Lab of Ornithology 2022). To supplement the *Carollia* focal data we collected, we also used published Phyllostomidae search-phase echolocation calls as a guide (Fig. 4.5 from Collen 2012). To avoid confusion among the cluttered environment and the presence of other bat species, we only noted calls above 40 kHz (Table S4; based on the typical minimum frequency of *Carollia* calls being approximately 45 kHz [Table S2]). Additionally, we only identified *Carollia* calls as such if they matched our focal data, consisted of at least two harmonics (to rule out the possibility that an FM call with one harmonic may be a different bat species altogether; see Fig. 3), and had a high signal-to-noise ratio on the main harmonic. The main harmonic was defined to be the harmonic with the highest relative amplitude (as seen in Fig. 1c, this would be the second harmonic for all three *Carollia* species).

### Diet and fruit scent data

As a third proxy of *Carollia-Piper* interactions, we compiled the percentage of *Piper* species (33 documented to date; 24 forest, 9 gap habitat) found in the respective diets of *C. castanea, C. sowelli*, and *C. perspicillata* at La Selva. These data were based on fecal samples collected from hundreds of free-ranging bats at La Selva and published by one of us (Santana et al. 2021, which incorporated data from Lopez and Vaughan 2007 and Maynard et al. 2019). For analyses, we calculated the maximum and average percentages of each *Piper* species present in the diet of all three *Carollia* species from this dataset (Table S5).

To investigate if fruit scent composition could be a potential factor explaining differences in *Carollia-Piper* interaction across ecotypes and habitats, we used a chemical dataset of *Piper* ripe fruit VOCs collected at La Selva and published by one of us (Santana et al. 2021). In that study, VOC emission data were obtained from ripe fruits for 21 *Piper* species via headspace adsorption methods and gas chromatography-mass spectrometry (GC-MS). Contaminants and all VOCs present in fewer than five samples were removed from that dataset, and GC-MS peaks were integrated and identified using the NIST 08 mass spectral library (see Santana et al. 2021, Supplementary Information).

To compare the fruit scent composition of forest against gap *Piper*, we classified all 21 species in the Santana et al. (2021) dataset into these ecotypes, for a total of 13 forest and 8 gap species. We sorted their total VOC emissions per weight for 249 VOCs for each species, resulting in a list of the most abundant chemicals in each species in order of highest to lowest concentration. We then took the first 20 chemicals in this sorted list for each species and combined them to find the most “common” chemical compounds among them (the VOCs present in the largest number of *Piper* species). This left us with 15 chemical compounds that are both present in sufficient amounts in *Piper*’s scent bouquet to potentially elicit an olfactory response (i.e., not trace amounts) and present in most of the *Piper* species in our dataset (avoiding zero values for our subsequent analyses). We used three chemical datasets in our statistical analyses: abundances of the 15 most common VOCs that we had compiled, total VOCs emission across all compounds, and total number of VOCs (Table S6).

### Statistical analyses

We performed all the statistical analyses in R v. 4.3.1 (R Core Team 2023). We tested for phylogenetic signals (Blomberg's K) in the chemical dataset using the time-calibrated, species-level *Piper* phylogeny published in Santana et al. (2021) and the function “physignal” in the package geomorph (Baken et al. 2021). To compare visitation and consumption across *Piper* ecotypes and habitats (open vs. gap; early-, mid-, and late-succession), we used Pearson's Chi-squared Test for Count Data and the function “chisq.test” (Pearson 1900) in the package stats (R Core Team 2023) with the argument to calculate Monte Carlo *P*-values set to “true” and using 2000 replicates in the Monte Carlo test to adjust for our small sample size (Hope 1968). The diet dataset consisted of proportions, so we linearized their sigmoid distribution by adding an arbitrary constant (c = 1) to avoid zero values, logit transformed (*y* = ln(p/(1-p)) the data (Armitage and Berry 1994) and performed Shapiro-Wilk's tests to test normality (Shapiro and Wilk 1965). These tests indicated that the transformed diet data for *C. castanea, C. sowelli*, and *C. perspicillata* followed normality (*W* = 0.4216, *P* = 9.047e-10; *W* = 0.42227, *P* = 2.789e-10; *W* = 0.54719, *P* = 6.164e-09), which was also the case for the maximum and average percentages of *Piper* in *Carollia* diets (*W* = 0.51649, *P* = 2.745e-09; *W* = 0.49507, *P* = 1.593e-09). We then performed analyses of variance (ANOVAs [Girden 1992]) to test for differences in the transformed percentages of *Piper* species (forest or gap, and early-, mid-, or late-succession) in *Carollia* diets. To test for differences in fruit scent between forest and gap *Piper*, we used the “nonpartest” function in the package nmpv (Burchett et al. 2017). This function calculates nonparametric relative effects for multivariate analyses of data that do not follow normality (data normality was tested with Shapiro-Wilk's) and returns test statistics with their permutation (randomization) analogs—we used the ANOVA global nonparametric test as described in Burchett et al. (2017).

## Results

### Patterns of *Carollia-Piper* interactions

The individual methods used to detect frugivores in relation to *Piper* plants had an influence on the type of information that could be retrieved about their interactions, and therefore, the conclusions that could be made about differences in frugivore communities between *Piper* ecotypes. At one end of the spectrum, passive acoustic recording data (in the form of identified echolocation calls) were informative of general bat activity and/or presence of bats near plants, whereas fecal samples directly collected from bats confirm whether this general bat activity includes fruit consumption that would lead to seed dispersal. Somewhere in between, camera trap video data provides information about plant visitation along with fruit exploratory and procurement behaviors (as *Carollia* do not feed at *Piper* plants directly but take the fruits to a feeding roost first [Wilson and Mittermeier 2019]). Below we describe how the data generated by these methods provides support for our hypothesis, or fails to do so. For more details about methodological considerations, see “*Notes on biomonitoring methods*” in the Supplementary Information.

#### Videos

Our camera traps allowed us to document *Carollia* collecting fruit at the forest species *P. cyanophyllum, P. generalense, P. nudifolium*, and *P. sublineatum*, and the gap species *P. colonense, P. reticulatum*, and *P. sancti-felicis* (Table 1, Supplementary Video 1). We observed *Carollia* visitations (flying by, inspecting fruits before leaving) at the forest species *P. generalense, P. nudifolium, P. paulowniifolium, P. sublinateum*, and the gap species *P. reticulatum, P. sancti-felicis*, and *P.* species D. Additionally, we were able to document *Piper* plant visitations and fruit consumptions by insects, birds, and small mammals other than bats (Table 1). Larger animals, such as tapirs, were recorded consuming entire *P. nudifolium* plants as they walked by. Rodents and possums were recorded passing by the cameras or climbing on the plants (F.V. pers. obs.; Supplementary Video 2). Birds would sometimes perch on the branches without consuming fruits. Based on this range of observations, we classified videos into different behaviors that involved *Piper* fruits: inspecting fruits, removing fruit, and eating fruit. We found bats and birds to most commonly take fruit off of the plants, although some birds ate the fruits while they remained attached to the plant (Supplementary Video 3). Fruit removal/consumption by non-bat frugivores only occurred at gap *Piper*, which were also consumed by birds and insects, whereas targeted collection of fruits by bats only occurred in forest *Piper*. This lends support to our initial hypothesis that frugivore diversity is dependent on *Piper* habitat.

We performed a chi-square test of independence (with computed *P*-values by Monte Carlo simulation due to small sample size) on the number of interactions between *Piper* plants and frugivores identified by camera traps against *Piper* ecotype (forest and gap) and failed to reject the null hypothesis that *Piper* ecotype has no effect on recorded frugivore diversity (*P* = 0.1999). However, a chi-square test of the same data but using the succession-based *Piper* habitat classification (early- and mid-succession; no late-succession *Piper* were recorded with camera traps or audiomoths) resulted in significant differences (*X*^2^ = 8, *df* = 1, *P* = 0.01799).

**Fig. 4:**
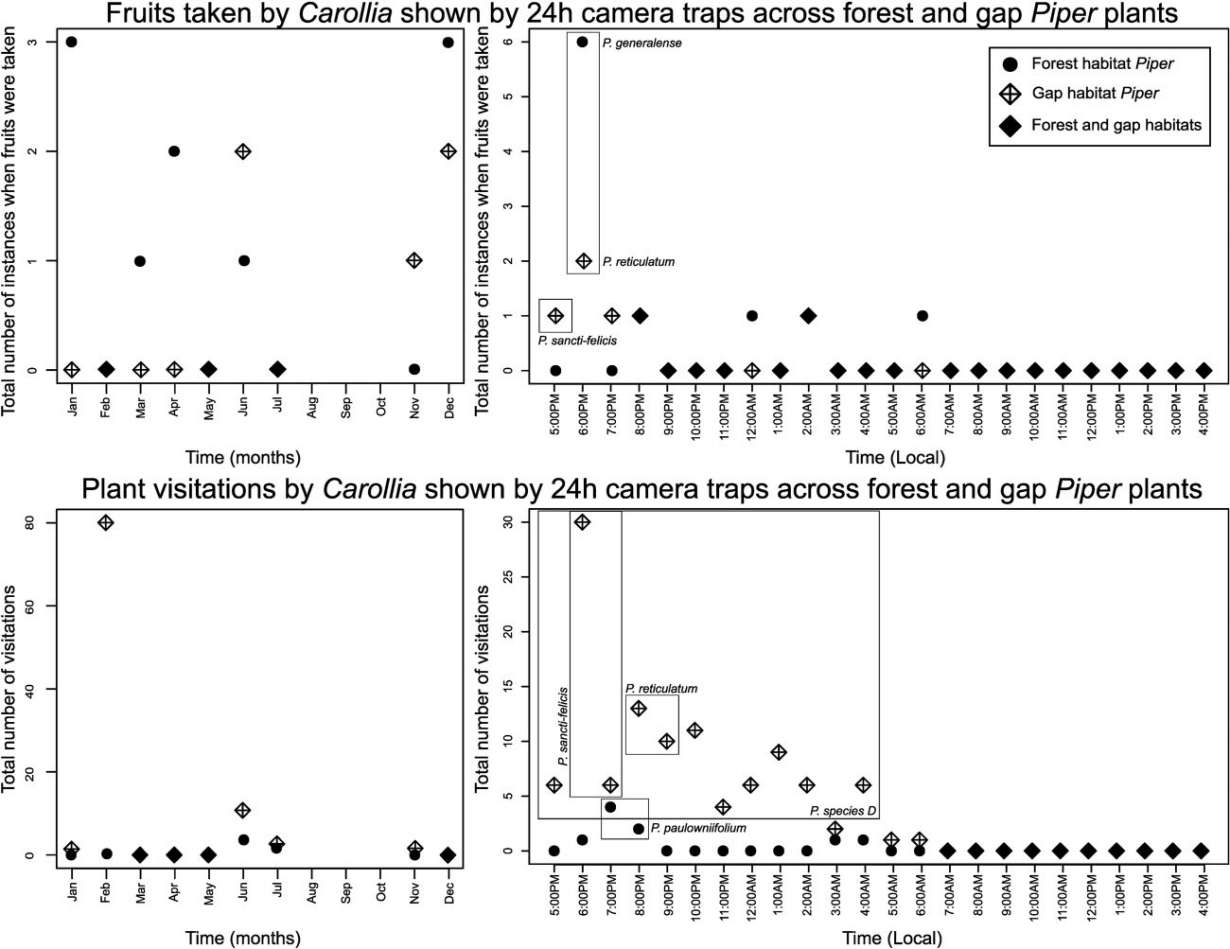
Temporal patterns of bat activity across forest and gap *Piper* plants. Top: Instances of bats taking fruits as shown by 24 h camera traps across forest and gap *Piper* over the course of the year and throughout the day. Data are shown starting from sunset (5:00 PM local time), when bat foraging begins. Bottom**:***Carollia* visitations to plants as shown by 24 h camera traps across the same temporal scales. High activity peaks are noted on the plots with the *Piper* species at which they occurred. Data are the visitations and instances of fruit acquisitions added across all plants of a species for a given month/hour throughout the length of the study.

**Fig. 5:**
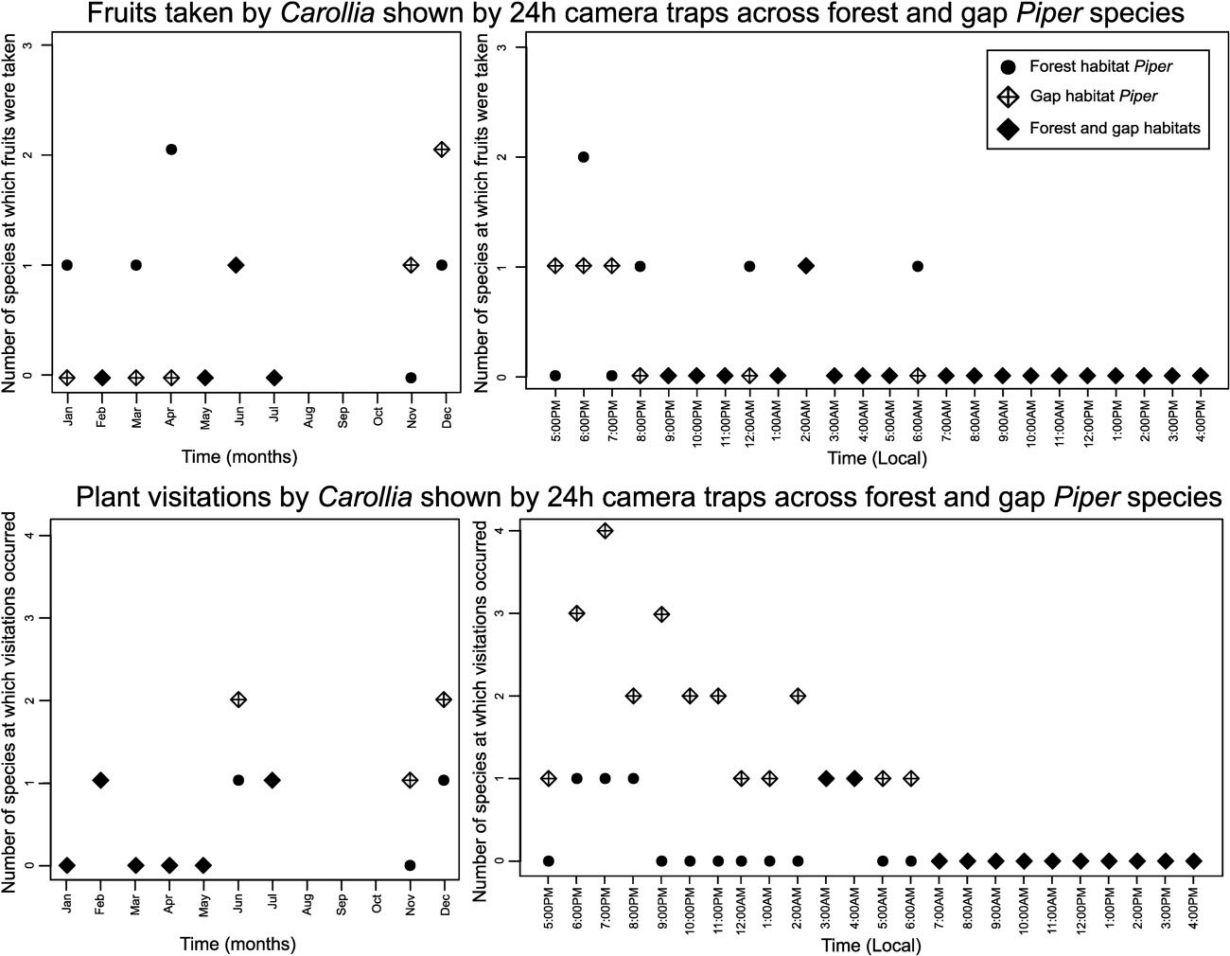
Temporal patterns of bat activity across forest and gap *Piper* species. Top: Total number of species at which instances of bats taking fruits occurred as shown by 24 h camera traps across forest and gap *Piper* over the course of the year and throughout the day. Data are shown starting from sunset (5:00 PM local time), when bat foraging begins. Bottom: Total number of species at which *Carollia* visitations to plants were recorded as shown by 24 h camera traps across the same temporal scales. Data are the visitations and fruit acquisitions recorded as a binary at each *Piper* species where camera data revealed bat activity, indicating the number of *Piper* species (classified by habitat) where activity was recorded at a certain time or during a month.

The camera trap data produced additional insight into the general activity patterns of *Carollia* visiting and consuming *Piper* over the course of the night and throughout the year. As seen in Fig. 4, general fruit acquisition and visitation activity by bats is continuous from dusk throughout the night until dawn, with a peak earlier in the night. We also documented more frequent visitations to gap *Piper* earlier in the night (Fig. 4) and observed a difference in the number of *Piper* species in which bat activity was recorded throughout the night (Fig. 5); bats visit a greater number of gap *Piper* species early in the night, and fewer species later on. This pattern was not seen at forest *Piper* plants, where bats visited a different forest *Piper* species every hour or so, but not more than one. Throughout the year (excluding August, September, and October, as we did not record field data during this time), we observed bats taking fruit from and visiting both the forest and gap *Piper*.

#### Acoustics

Acoustic monitoring allowed us to document the presence of bats at the forest *Piper* species *P. generalense, P. nudifolium*, and *P. paulowniifolium*, and the gap species *P. reticulatum*. Collection of *Piper* fruits by bats could not be identified purely by this method. However, we identified search-phase echolocation calls with harmonics, which indicate *Carollia* bats flying by, if not visiting *Piper* plants to inspect fruits. We performed chi-square tests of independence on the acoustic visitation results with *Piper* ecotype and habitat (open, forest, or early-, mid-, or late-succession, respectively) as predictor variables; the relationship between these two variables was not significant in both cases (*P* = 0.2124; *P* = 1).

#### Diet

We used ANOVAs to compare the maximum and average percentages of *Piper* in *Carollia* diets against the ecotype and habitat classifications as predictor variables. These analyses resulted in statistically significant differences in consumption of *Piper* species (ecotype- and succession-category schemes; *P* < 0.05, see Table 2). The results further provide evidence that all three *Carollia* species consume significantly more gap (early-succession) *Piper* than forest (mid-, late-succession) *Piper*. Nonparametric inference for the comparison of multivariate data samples (Burchett et al. 2017) testing the aforementioned variables indicated that there is a 95% probability that a randomly chosen *Carollia* would exhibit a larger percentage of gap than forest *Piper* in their diet.

**Table 2:** One-way analyses of variance (ANOVAs) testing the % of *Piper* species in the diets of the three *Carollia* species against habitat as a predictor variable (following logit transformation and testing for normality).

	*df*	Sum sq.	Mean sq.	*F*-value	*P*
Predictor variable: ecotype (forest, gap)					
*C. castanea*	1	0.04763	0.04763	14.08	0.000725***
*C. sowelli*	1	0.06412	0.06412	13.63	0.000852***
*C. perspicillata*	1	0.05261	0.05261	23.76	0.000031***
Max. %	1	0.09305	0.09305	19.63	0.000109***
Average %	1	0.05363	0.05363	18.38	0.000164***
	*df*	Sum sq.	Mean sq.	*F*-value	*P*
Predictor variable: Succession habitat (early, mid, late-succession)					
*C. castanea*	2	0.05356	0.026782	8.119	0.001520**
*C. sowelli*	2	0.06858	0.03429	7.279	0.002650**
*C. perspicillata*	2	0.05534	0.27671	12.59	0.000107***
Max. %	2	0.1037	0.05183	11.41	0.000207***
Average %	2	0.05772	0.028861	10.03	0.000463***

** *P* < 0.05.

*** *P* < 0.001.

### Fruit scent composition as a medium for interpreting *Carollia-Piper* habitat patterns

**Fig. 6:**
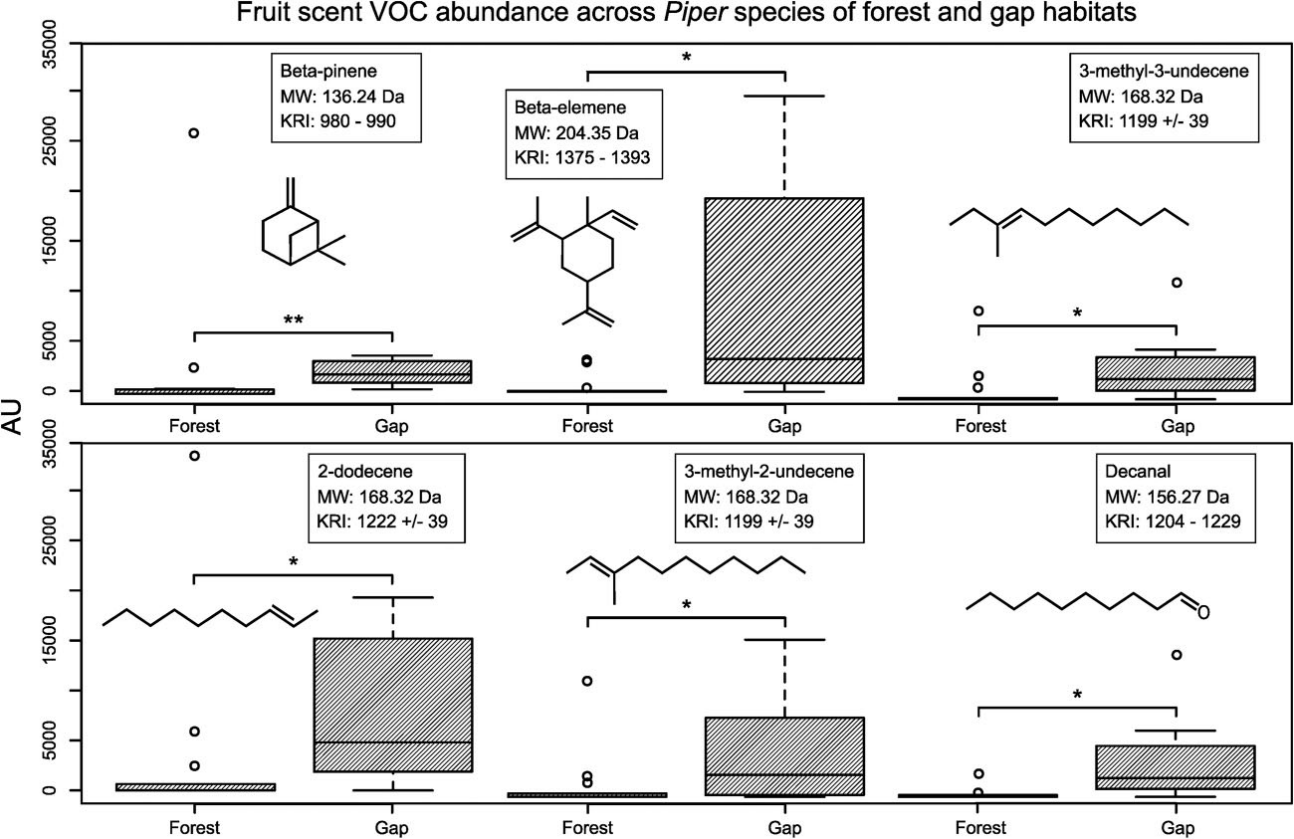
Six chemicals that are significantly different between gap and forest *Piper* fruit scent compositions (beta-pinene, beta-elemene, 3-methyl-3-undecene, 2-dodecene, 3-methyl-2-undecene), showing the difference in VOC emissions per weight between forest and gap species (*P < 0.05, **P < 0.01). For reference, chemical structures, molecular weights (MW), and Kovats Retention Indices (KRI) are included for each chemical. KRIs for 2-dodecene, 3-methyl-2-undecene, and 3-methyl-3-undecene were referenced from ChemSpider. KRIs for beta-pinene, beta-elemene, and decanal were reported as ranges for column type DB-5 (matched with the methods reported in Santana et al. 2021) collected from the literature sources compiled in The Pherobase: Database of Pheromones and Semiochemicals.

The variation in the fruit scent VOC data used in our analyses was not highly impacted by the evolutionary relationships between *Piper* species; there was no significant phylogenetic signal for almost all of the first 15 most common VOCs, with the exception of the most common VOC across the *Piper* species in the dataset, alpha-caryophyllene, which approached significance (Blomberg's *K* = 0.83, *P* = 0.053). Results from nonparametric multivariate tests indicated significant differences in the chemical composition of forest vs. gap *Piper* (test statistic = 3.126, *df*_1_ = 6.004, *df*_2_ = 106.1721, *P* = 0.007, permutation test *P* = 0.006). Pairwise comparisons using a Wilcoxon rank sum test (Wilcoxon 1945) with continuity correction (*P*-value adjustment method: Benjamini and Hochberg [Benjamini and Hochberg 1995]) yielded significant differences between gap vs. forest *Piper* (Fig. 6) for the VOCs beta-pinene (*P* = 0.0052), 2-dodecene (*P* = 0.046), beta-elemene (*P* = 0.011), 3-methyl-2-undecene (*P* = 0.038), 3-methyl-3-undecene (*P* = 0.038), and decanal (*P* = 0.014). We did not find differences in the total emission and number of VOCs among *Piper* species classified by ecotype (*P* = 0.244; *P* = 0.153) or habitat succession stage (*P* = 0.663; *P* = 0.074).

## Discussion

### *Piper-Carollia* interactions vary across ecotypes and habitats

Our study aimed to document and contrast frugivore visitation patterns across putative forest and gap *Piper* ecotypes in Costa Rica to gain more insight into the *Carollia-Piper* mutualism, and henceforth allow for a better understanding of biodiversity and behavioral ecology in the Neotropics. We integrated three biomonitoring modalities to devise the best approach to characterize these fruit-bat interactions and interpret the relationship between Neotropical *Piper* and *Carollia* bats. Our analysis of nightly acoustic recordings allowed us to identify *Carollia* activity at or near *Piper* plants; however the number of *Carollia* visitations found by our acoustic analyses was not sufficiently large for determining differences in bat presence at one *Piper* ecotype or habitat vs. another. Video-based camera traps provided a better understanding of interactions between *Carollia* and *Piper*: by examining video recordings, we were able to directly see *Carollia* taking *Piper* fruit and *Carollia* inspecting fruit for some time before grabbing one (or not) and flying away. We were also able to see a variety of other animals interacting with *Piper* plants, providing a unique “plant perspective” of the interaction. Due to this functionality of the camera traps, we were able to find support for the hypothesis that forest *Piper* species depend on *Carollia* as their main seed dispersers during their shorter fruiting periods, whereas gap species exhibit a broader range of dispersers, most of which are still bats (Table 1). It is important to note, however, that we found statistical support for these differences only when *Piper* were classified into habitat categories (early-, mid-, late-succession). This discrepancy may have resulted from the fact that many mid-succession *Piper* species do not fall neatly in a forest vs. gap categorization, but rather in between.

The results of diet data analyses were consistent with those from video data in uncovering significant differences in *Carollia* interactions across *Piper species* of different habitats, even at the ecotype level. By capitalizing on annual diet data based on fecal samples collected from hundreds of bats, we were able to describe that *Carollia* (*C. sowelli, C. perspicillata, C. castanea*) consume a greater percentage of gap *Piper* species than forest *Piper* species. As proposed by Thies and Kalko (2004), phenology can provide an explanation for this phenomenon; forest *Piper* species produce fruit for a relatively shorter time period than gap *Piper*, and therefore gap *Piper* consumption will be higher on average when considered throughout the year. This may not be necessarily indicative of preference for one ecotype over the other, however; cross-checking with visitation or consumption data on smaller temporal scales (e.g., during the same night across species and habitats), as can be done by camera trap data analysis, could help gain more insights regarding the finer scale dynamics of the *Carollia-Piper* mutualism. At present, our camera trap data are not sufficient to do so, since there were few bat visits/consumption events that coincided between the forest and gap *Piper* at the same time interval during the night. However, our camera results do provide some evidence that *Carollia* could be visiting and consuming a greater number of *Piper* species –particularly gap species– earlier in the night, followed by decreased activity and a switch to consumption of forest *Piper* later in the night (Figs. 4 and 5). These results are consistent with the findings of Heithaus and Fleming (1978), who noted *C. perspicillata* activity throughout the night, and highlighted possible preferences for gap *Piper* species and opportunities for temporal resource partitioning. Future studies could use the methods presented here across a greater number of plants in selected *Piper* species to look more closely at these patterns.

### Differences in *Piper* scent volatiles may influence *Carollia-Piper* interactions across habitats

In order to mechanistically understand *Carollia-Piper* interactions, our second aim was to examine the differences between forest and gap *Piper* in terms of their fruit scent chemical composition. In particular, the extent of ripeness and the type and strength of the chemical cues could be key to affecting bat visitations and behaviors, as a ripe fruit with a strong signal could be located and seized very quickly, whereas a fruit still ripening or with weak signals may end up uneaten even after a long period of inspection. We find evidence for differences in the chemical composition of fruit scent between forest and gap *Piper* species (Fig. 6); via olfactory preferences, these distinguishing chemicals could potentially underlie differences in *Carollia* visitation and consumption to and of *Piper* across habitats. We identified six VOCs among the most common chemicals found in the scent bouquet of 21 *Piper* species to be significantly more abundant in gap *Piper* compared to forest *Piper*. Two are terpenes (one monoterpene, one sesquiterpene), three are long hydrocarbon (C_n_ = 10, C_n_ = 11) chain alkenes, and one is a long hydrocarbon chain (C_n_ = 10) aldehyde. Studies have shown that mammals, including bats, have a higher olfactory performance (sensitivity) when tested on compounds containing longer carbon chains (Laska et al. 2000); hence, even low concentrations of these compounds in the scent bouquets of *Piper* are likely to attract bats to the fruit (Borges et al. 2008). These findings become especially relevant when we consider that gap *Piper* fruits may ripen during the day and therefore be exposed to higher temperatures and more sunlight than the ripe fruits of forest *Piper;* these abiotic factors may affect the distance to which VOC emissions travel and are detectable by *Carollia's* olfaction. Our results (long hydrocarbon chain compounds found to be more abundant in the emissions of gap *Piper* fruits compared to forest) provide further evidence that *Piper* fruit scents may influence *Carollia* visitations across habitats.

Santana et al. (2021) found that highly consumed *Piper* species, which are included in our dataset, are phylogenetically scattered and characterized by scents rich in terpenoids, similar to other bat-dispersed fruits (which contain high abundances of monoterpenes [Hodgkison et al. 2013; Santana et al. 2021]). Our results, which include beta-pinene and beta-elemene (terpenoids characteristic to gap *Piper* species), support these findings. Importantly, the fruit scent of a *Piper* species highly consumed by *Carollia* (*P. sancti-felicis*) is also unique in containing 2-heptanol, an aliphatic alcohol preferred by *Carollia* in behavioral experiments (Leiser-Miller et al. 2020; Santana et al. 2021). Thus, particular notes in the fruit scent bouquet may also play a role in *Piper* preferences. We did not consider unique scent notes in small proportions for our broader-scale analysis—we focused on chemicals presenting relatively large abundance in nearly all *Piper* species in our dataset. Thus, further behavioral experiments are necessary to determine if and which fruit scent chemicals contribute to driving differences in bat foraging behavior and *Piper* consumption across habitats; the aforementioned terpenoids and hydrocarbon chain compounds we identified through our analyses are good candidates for this future work.

## Conclusion

Our results integrating acoustic, camera trap, and diet data lend support to the hypothesis that forest and gap *Piper* differ in their diversity of interacting frugivores, with forest *Piper* exhibiting a tight relationship with bats, and gap *Piper* interacting with a wider spectrum of frugivores. We found that, for the three *Carollia* species present at our study site (*C. sowelli, C. perspicillata, C. castanea*), gap *Piper* was consumed significantly more than forest *Piper*, but visitations and fruit acquisition by bats occurred across both forest and gap *Piper* throughout the year, and forest *Piper* were only visited by *Carollia*. Therefore, the *Carollia-Piper* mutualism hinges on the regular ingestion of *Piper* fruit by *Carollia* in tandem with variation in the fruiting peaks roughly characteristic to gap and forest *Piper*; forest *Piper* species rely on *Carollia* for seed dispersal during their short fruiting period, and gap *Piper* provide nutrients year-round for *Carollia*. We observed *Carollia* visitations to gap and forest *Piper* throughout the night, and further found evidence for a foraging activity peak closer to dusk characterized by a greater variety of gap *Piper* species visited or collected by bats. By incorporating fruit scent chemical data into our analyses of *Piper* ecotypes, we not only find support for the hypothesis that scent signals might drive differential foraging by *Carollia* on *Piper* fruits, but we identify specific compounds (terpenoids, hydrocarbon chain derivatives) that may influence *Carollia* visitations across forest and gap habitats. Our study highlights the benefit of integrating multiple biomonitoring methods and datasets to characterize plant-animal interactions.

## Supplementary Material

obae028_Supplemental_Files

## Data Availability

The data underlying this article are available in the article and in its online supplementary material.

## References

[bib1] Baken E , Collyer M, Kaliontzopoulou A, Adams D. 2021. geomorph v4.0 and gmShiny: enhanced analytics and a new graphical interface for a comprehensive morphometric experience Methods Ecol Evol 12:2355–63.

[bib2] Armitage P , Berry G. 1994. Statistical methods in medical research. 3rd ed., Blackwell Scientific Publications, Oxford.

[bib3] Barré K , Le Viol I, Julliard R, Pauwels J, Newson SE, Julien JF, Claireau F, Kerbiriou C, Bas Y. 2019. Accounting for automated identification errors in acoustic surveys. Methods Ecol Evol 10:1171–88.

[bib4] Benjamini Y , Hochberg Y. 1995. Controlling the false discovery rate: a practical and powerful approach to multiple testing. J R Stat Soc Series B Stat Methodol 57:289–300.

[bib5] Borges RM , Bessière JM, Hossaert-McKey M. 2008. The chemical ecology of seed dispersal in monoecious and dioecious figs. Funct Ecol 22:484–93.

[bib6] Burchett WW , Ellis AR, Harrar SW, Bathke AC. 2017. Nonparametric inference for multivariate data: the R package npmv. J Stat Softw 76:1–18.36568334

[bib7] Collen A . 2012. The evolution of echolocation in bats: a comparative approach. Doctoral thesis, University College London.

[bib8] Fleming TH . 1991. The relationship between body size, diet, and habitat use in frugivorous bats, genus Carollia (Phyllostomidae). J Mammal 72:493–501.

[bib9] Fraser EE , Silvis A, Brigham RM, Czenze ZJ. 2020. Bat echolocation research: a handbook for planning and conducting acoustic studies (2nd ed.). Bat Conservation International, United States of America.

[bib10] Garrigues R , Dean R. 2007. The birds of Costa Rica: a field guide. Cornell University Press, United States of America.

[bib11] Gelambi M , Whitehead SR. 2023. Multiscale variability in nutrients and secondary metabolites in a bat-dispersed neotropical fruit. Ecol Evol 13:e10453. 37664504 10.1002/ece3.10453 PMC10474796

[bib12] Girden ER . 1992. ANOVA: repeated measures (no. 84). Sage.

[bib13] Greig N . 1993. Regeneration mode in neotropical Piper: habitat and species comparisons. Ecology 74:2125–35.

[bib14] Heithaus ER , Fleming TH. 1978. Foraging movements of a frugivorous bat, *Carollia perspicillata* (Phyllostomatidae). Ecol Monogr 48:127–43.

[bib15] Hodgkison R , Ayasse M, Häberlein C, Schulz S, Zubaid A, Mustapha WAW, Kunz TH, Kalko EK. 2013. Fruit bats and bat fruits: the evolution of fruit scent in relation to the foraging behaviour of bats in the new and old World tropics. Funct Ecol 27:1075–84.

[bib16] Hope ACA . 1968. A simplified Monte Carlo significance test procedure. J R Stat Soc Ser B 30:582–98.

[bib17] Jones G , Jacobs DS, Kunz TH, Willig MR, Racey PA. 2009. Carpe noctem: the importance of bats as bioindicators. Endangered Species Res 8:93–115.

[bib19] Kalko EK , Condon MA. 1998. Echolocation, olfaction and fruit display: how bats find fruit of flagellichorous cucurbits. Funct Ecol 12:364–72.

[bib20] Kunz TH , Braun de Torrez E, Bauer D, Lobova T, Fleming TH. 2011. Ecosystem services provided by bats. Ann N Y Acad Sci 1223:1–38. 21449963 10.1111/j.1749-6632.2011.06004.x

[bib21] Laska M , Seibt A, Weber A. 2000. ‘Microsmatic’ primates revisited: olfactory sensitivity in the squirrel monkey. Chem Senses 25(1):47–53. 10667993 10.1093/chemse/25.1.47

[bib22] Leiser-Miller LB , Kaliszewska ZA, Lauterbur ME, Mann B, Riffell JA, Sharlene SE. 2020. A fruitful endeavor: scent cues and echolocation behavior used by *Carollia castanea* to find fruit. Integr Org Biol 2:obaa007. 33791551 10.1093/iob/obaa007 PMC7671165

[bib18] Lisa K . 2022. Raven pro: interactive sound analysis software (Version 1.6.2). Ithaca, NY: Yang Center for Conservation Bioacoustics at the Cornell Lab of Ornithology, The Cornell Lab of Ornithology.

[bib24] Lopez JE , Vaughan C. 2007. Food niche overlap among neotropical frugivorous bats in Costa Rica. Rev Biol Trop 55:301–13. 18457139

[bib25] Luo B , Leiser-Miller L, Santana SE, Zhang L, Liu T, Xiao Y, Liu Y, Feng J. 2019. Echolocation call divergence in bats: a comparative analysis. Behav Ecol Sociobiol 73: 154.

[bib26] Maynard LD , Ananda A, Sides MF, Burk H, Whitehead SR. 2019. Dietary resource overlap among three species of frugivorous bat in Costa Rica. J Trop Ecol 35:165–72.

[bib27] Mello MAR , Felix GM, Pinheiro RBP, Mulaert RL, Geiselman C, Santana SE, Tschapka M, Lotfi N, Rodrigues FA, Stevens RD. 2019. Insights into the assembly rules of a continent-wide multilayer network. Nat Ecol Evol 3:1525–32. 31611677 10.1038/s41559-019-1002-3

[bib28] OTS . 2023. Organization for tropical studies database: florula digital de la selva. See https://sura.ots.ac.cr/florula4/ Accessed September 14, 2023.

[bib29] Pearson K . 1900. X. On the criterion that a given system of deviations from the probable in the case of a correlated system of variables is such that it can be reasonably supposed to have arisen from random sampling. London Edinburgh Dublin Philos Mag J Sci 50:157–75.

[bib30] Pettersson Elektronik AB . 2016. BatSound Version 4.4. Uppsala, Sweden.

[bib31] R Core Team . 2023. R: a language and environment for statistical computing. R Foundation for Statistical Computing. Vienna, Austria.

[bib32] Russo D , Ancillotto L, Jones G. 2017. Bats are still not birds in the digital era: echolocation call variation and why it matters for bat species identification. Can J Zool 96:63–78.

[bib33] Russo D , Voigt CC. 2016. The use of automated identification of bat echolocation calls in acoustic monitoring: a cautionary note for a sound analysis. Ecol Indic 66:598–602.

[bib34] Rydell J , Nyman S, Eklöf J, Jones G, Russo D. 2017. Testing the performances of automated identification of bat echolocation calls: a request for prudence. Ecol Indic 78:416–20.

[bib35] Santana SE , Kaliszewska ZA, Leiser-Miller LB, Lauterbur ME, Arbour JH, Dávalos LM, Riffell JA. 2021. Fruit odorants mediate co-specialization in a multispecies plant–animal mutualism. Proc R Soc B 288:20210312. 10.1098/rspb.2021.0312 PMC8354748 34375556

[bib36] Schwab IR , Pettigrew J. 2005. A choroidal sleight of hand. Br J Ophthalmol 89:1398–. 16267906 10.1136/bjo.2005.077966 PMC1772916

[bib37] Shapiro SS , Wilk MB. 1965. An analysis of variance test for normality (complete samples). Biometrika 52:591–611.

[bib38] The MathWorks Inc . 2022. MATLAB Version: 9.13.0 (R2022b), Natick, MA: The MathWorks Inc.

[bib39] Thies W , Kalko E, Schnitzler HU. 1998. The roles of echolocation and olfaction in two neotropical fruit-eating bats, *Carollia perspicillata* and *C. castanea*, feeding on Piper. Behav Ecol Sociobiol 42:397–409.

[bib40] Thies W , Kalko EK. 2004. Phenology of neotropical pepper plants (Piperaceae) and their association with their main dispersers, two short-tailed fruit bats, *Carollia perspicillata* and *C. castanea* (Phyllostomidae). Oikos 104:362–76.

[bib41] von Helversen D , von Helversen O. 1999. Acoustic guide in bat-pollinated flower. Nature 398:759–60.

[bib42] Whelan CJ , Wenny DG, Marquis RJ. 2008. Ecosystem services provided by birds. Ann N Y Acad Sci 1134:25–60. 18566089 10.1196/annals.1439.003

[bib43] Wilcoxon F . 1945. “Individual comparisons by ranking methods.” Biometr Bull 1:80–3.

[bib44] Wilson DE , Mittermeier RA. 2019. Phyllostomidae. In: Handbook of the mammals of the world—Volume 9 Bats. Barcelona: Lynx Edicions: 444–583, ISBN: 978-84-16728-19-0.

