# Supplementary material for: Effects of Habitat and Fruit Scent on the Interactions between Short-tailed Fruit Bats and Piper Plants
Source: Integr Org Biol. 2024 Jul 29;6(1):obae028. doi: 10.1093/iob/obae028 (PMC11316396; doi:10.1093/iob/obae028)
Supplement: obae028_Supplemental_Files [file obae028_supplemental_files.zip › Supplementary Information FINAL.docx]

**Supplementary Information for:**

**Effects of habitat and fruit scent on the interactions between short-tailed fruit bats and *Piper* plants**

Sneha Sil, Florencia Visconti, Gloriana Chaverri, Sharlene E. Santana

Supplementary materials

Notes on biomonitoring methods

Supplementary Video 1: Bat flying past *Piper* plant and acquiring fruit.

Supplementary Video 2: Mouse climbing on a *Piper generalense* plant.

Supplementary Video 3: Passerini’s tanager consuming *Piper* *sancti-felicis*.

Supplementary tables

**S1.** Recorder locations, plant site locations, number of ripe fruits, starting and end times/dates

**S2.** Focal call parameters from search-phase echolocation calls of three *Carollia* species

**S3.** Number of acoustic files identified as containing bat calls via acoustic algorithm

**S4.** Number of each type of bat echolocation call identified within the acoustic dataset

**S5.** Diet dataset from the literature (Santana et al., 2021; Lopez & Vaughan, 2007; Maynard et al., 2019) with *Piper* habitat classifications

**S6.** Chemical dataset sorted by concentrations per *Piper* species of the 15 most abundant chemicals among all *Piper* species published in Santana et al. 2021

**Notes on biomonitoring methods**

The integration of biomonitoring methods in our study had significant benefits to document *Piper*-frugivore interactions, but also had important limitations and challenges. We share some of these here with the aim of providing details that could help understand the results of this study, as well as inform future work:

*Acoustic recordings*

*Carollia* have low-intensity calls characteristic of phyllostomids, therefore our acoustic recorders were turned up to high gain, leading to a high level of background noise in the recordings. After filtering, we obtained clear evidence of general bat activity at most of the *Piper* species and plants, which ruled out the possibility of our recorders failing to detect ultrasonic signals. These results were dominated by insectivorous bats in the vicinity of *Piper* plants. These calls were identified by the presence of FM-QCF (frequency-modulated, quasi-constant frequency), FM-CF (frequency-modulated, constant frequency), FM-CF-FM (frequency-modulated, constant frequency, frequency-modulated) calls and feeding/terminal buzzes (see Fig. 3, Table S4). Additionally, we found numerous calls of the common vampire bat (*Desmodus rotundus*) in our data due to the presence of a large roost near one of the sites (S. Sil personal observation). The key differences between the calls of *Carollia* and insectivorous bats’ are that *Carollia*’s calls exhibit higher frequencies and purely frequency-modulated components. *Carollia* and *D. rotundus* calls were distinguished by their higher and lower frequency bandwidths, respectively (Collen 2012). However, classification of *Carollia* calls down to the lowest taxonomic level (species) was unreliable due to both the intraspecific and interspecific variability in type of call (social, search-phase), diagnostic call traits, and similar call structure to other Phyllostomidae species (Obrist 1995, Barclay 1999, Russo et al. 2018, Fraser et al. 2020, Fig. 4.5 in Collen 2012). Identification to the species level is especially difficult for low-duty cycle call species such as *Carollia* spp., because their calls exhibit the most intraspecific and intraindividual flexibility associated with different tasks and habitat effects (Russo et al. 2018).

Given that *Carollia* are some of the most abundant bats at La Selva, we believe technical challenges probably affected our ability to successfully record clear *Carollia* search-phase echolocation calls. In particular, the scarcity of *Carollia* echolocation calls found in the passive acoustic monitoring field data compared to the abundance of detected insectivorous bat activity directed us to look into additional physical factors that could have affected these results. Bat species that emit broad bandwidth calls like *Carollia* are more successful in detecting details and therefore finding food items in cluttered environments, but the high frequencies that dominate these FM calls undergo significant atmospheric attenuation, so these calls are only effective and detectable by acoustic devices over a short range (Griffin 1971; Lawrence and Simmons 1982; Neuweiler 1990). *Carollia* calls are low intensity and high frequency, and attenuate very quickly in warm, humid environments (e.g., 45–90 kHz attenuates at 1.4–4 dB/m at 25ºC and 80% humidity [Jakobsen et al. 2013; Leiser-Miller et al. 2020]). To provide further support for this phenomenon, we did not detect any calls above 70-80kHz in our recordings (S. Sil personal observation). Bats also tend to increase call frequency and pulse rates and decrease the duration of their calls to avoid forward masking, which is essential in cluttered environments and dense vegetation (Schnitzler and Kalko 2001). In summary, distance, clutter, and noise all affect the quality of acoustic recordings in the field (Fraser et al. 2020); it is up to the discretion of the scientists wishing to monitor bat activity in their chosen field site to decide at which taxonomic level they are comfortable identifying (based on the available call libraries and focal data), how they will filter the noise from their recordings, and how to account for phenomena such as atmospheric attenuation in their results.

*Camera traps*

Camera traps are considered a minimally invasive surveying method when compared to those requiring capture or human presence, such as mist-netting or visual counts (Sollmann et al. 2013; Krivek et al. 2021). However, bats, due to their small size, nocturnal habits, and ability to fly quickly (Krivek et al. 2021) have been a challenge to monitor with traditional methods like photo-based camera traps. In our study, and thanks to technical advances in detection speed and infrared recording, we were able to successfully document bat and other frugivore visits to *Piper* plants. However, combing through thousands of hours’ worth of videos is still an arduous task (automated methods proved inefficient due to wind- or rain-caused plant movement and other environmental factors) and may not provide the exact information needed to characterize some bat-plant interactions. For example, in our system, some interactions were never detected despite the *Piper* species being reported as one significant component of *Carollia’s* diet (e.g., gap species *P. umbricola;* average 8.51% of the diet of three *Carollia* species [Santana et al. 2021]). While the reasons why the bats never visited the selected plants of these species during our study are not clear, this issue could be solved by increasing the sample size and duration of the study.

*Diet data*

Examining which *Piper* species are present in fecal samples collected in the field is the most direct way to measure consumption of *Piper* by *Carollia*. The results of diet data analyses were consistent with those from video data in uncovering significant differences in *Carollia* interactions across *Piper* of different habitats, even at the coarser levels of habitat classification. This suggests that the more intensive and direct approach of collecting fecal samples brings us closer to discovering habitat effects in this *Carollia-Piper* mutualism. However, it is important to consider that analyses of bat fecal samples for dietary identification at this scale is labor-intensive both in the field and the lab and requires seed reference libraries collected over months or years.

Overall, our results indicate that analyzing diet datasets synergizes well with real-time monitoring of plants and bat activity, however; by combining acoustic and video methods, we gained insight into plant-bat interactions across different levels: bat presence around plants, visitations to plants, inspection of plants, and acquisition and consumption of fruits.

**Table 1.** Information on the *Piper* plants studied in the field, their locations, the dates and number of days recorded (AudioMoths and camera traps).

| **Species** | **Habitat**  **Classification** | **Site** | **# ripe fruits** | **Duration (days)** | **Start date** | **End date** | **LAT (N)** | **LONG (W)** |
| --- | --- | --- | --- | --- | --- | --- | --- | --- |
| *P. auritum* | gap; early-suc | 1 | 2 | 36 | 6/5/2021 | 7/11/2021 | 10.43146 | -84.00369 |
| *P. colonense* | gap; mid-suc | 1 | 3 | 12 | 12/1/2019 | 12/13/2019 | 10.4308 | -84.00645 |
|  |  | 2 | 4 | 11 | 6/4/2021 | 6/15/2021 | 10.43089 | -84.00334 |
|  |  | 3 | 3 | 22 | 6/15/2021 | 7/7/2021 | 10.43015 | -84.00971 |
| *P. cyanophyllum* | forest; mid-suc | 1 | 1 | 139 | 1/26/2020 | 6/13/2020 | 10.4311 | -84.00666 |
| *P. generalense* | forest; mid-suc | 1 | 3 | 84 | 11/16/2019 | 2/8/2020 | 10.42501 | -84.0016 |
|  |  | 2 | 2 | 84 | 11/16/2019 | 2/8/2020 | 10.42502 | -84.00171 |
|  |  | 3 | 5 | 70 | 11/16/2019 | 1/25/2020 | 10.42502 | -84.0017 |
|  |  | 4 | 2 | 14 | 11/16/2019 | 11/30/2019 | 10.42483 | -84.00164 |
|  |  | 5 | 2 | 70 | 11/16/2019 | 1/25/2020 | 10.43071 | -84.00674 |
|  |  | 6 | 3 | 96 | 11/17/2019 | 2/21/2020 | 10.4335 | -84.01216 |
|  |  | 7 | 7 | 211 | 11/17/2019 | 6/15/2020 | 10.43377 | -84.01219 |
|  |  | 8 | 14 | 135 | 1/26/2020 | 6/9/2020 | 10.43733 | -84.00581 |
| *P. multiplinervium* | gap; early-suc | 1 | 5 | 69 | 11/16/2019 | 1/24/2020 | 10.4306 | -84.00658 |
| *P. nudifolium* | forest; mid-suc | 1 | 3 | 140 | 1/27/2020 | 6/15/2020 | 10.43543 | -84.03122 |
| *P. paulowniifolium* | forest; mid-suc | 1 | 7 | 35 | 6/6/2021 | 7/11/2021 | 10.4328 | -84.00667 |
|  |  | 2 | 11 | 35 | 6/6/2021 | 7/11/2021 | 10.43854 | -84.01363 |
|  |  | 3 | 22 | 25 | 6/16/2021 | 7/11/2021 | 10.43749 | -84.01147 |
| *P. reticulatum* | forest; mid-suc | 1 | 18 | 44 | 11/17/2019 | 12/31/2019 | 10.43821 | -84.01594 |
|  |  | 2 | 16 | 16 | 11/27/2019 | 12/13/2019 | 10.43045 | -84.0059 |
|  |  | 3 | 14 | 12 | 6/3/2021 | 6/15/2021 | 10.43699 | -84.01009 |
|  |  | 4 | 3 | 12 | 6/3/2021 | 6/15/2021 | 10.43642 | -84.00952 |
|  |  | 5 | 6 | 6 | 6/4/2021 | 6/10/2021 | 10.43295 | -84.00443 |
|  |  | 6 | 4 | 9 | 6/6/2021 | 6/15/2021 | 10.43446 | -84.00821 |
|  |  | 7 | 3 | 13 | 6/10/2021 | 6/23/2021 | 10.43053 | -84.00675 |
|  |  | 8 | 5 | 8 | 6/10/2021 | 6/18/2021 | 10.43015 | -84.00971 |
|  |  | 9 | 11 | 25 | 6/16/2021 | 7/11/2021 | 10.43069 | -84.00661 |
|  |  | 10 | 9 | 18 | 6/23/2021 | 7/11/2021 | 10.42969 | -84.00836 |
| *P. sanctifelicis* | gap; early-suc | 1 | 2 | 3 | 11/16/2019 | 11/19/2019 | 10.43115 | -84.00694 |
|  |  | 2 | 2 | 5 | 11/19/2019 | 11/24/2019 | 10.431 | -84.00646 |
|  |  | 3 | 2 | 3 | 11/24/2019 | 11/27/2019 | 10.43076 | -84.00656 |
|  |  | 4 | 1 | 1 | 12/1/2019 | 12/2/2019 | 10.43043 | -84.00608 |
|  |  | 5 | 1–3 | 8 | 12/5/2019 | 12/13/2019 | 10.43075 | -84.00682 |
|  |  | 6 | 3 | 9 | 1/27/2020 | 2/5/2020 | 10.43005 | -84.01115 |
|  |  | 7 | 3 | 17 | 2/5/2020 | 2/22/2020 | 10.43084 | -84.00675 |
|  |  | 8 | 7 | 5 | 2/12/2020 | 2/17/2020 | 10.43148 | -84.00584 |
|  |  | 9 | 1 | 4 | 6/6/2021 | 6/10/2021 | 10.43136 | -84.00648 |
|  |  | 10 | 20+ | 23 | 6/18/2021 | 7/11/2021 | 10.42989 | -84.00835 |
|  |  | 11 | 6 | 13 | 6/28/2021 | 7/11/2021 | 10.43322 | -84.00737 |
| *P. species* D | gap; mid-suc | 1 | 6+ | 31 | 1/26/2020 | 2/26/2020 | 10.43052 | -84.00613 |
|  |  | 2 | 3 | 16 | 2/10/2020 | 2/26/2020 | 10.43132 | -84.0061 |
|  |  | 3 | 4 | 12 | 6/15/2021 | 6/27/2021 | 10.43104 | -84.00624 |
| *P. sublinateum* | forest; mid-suc | 1 | 2 | 40 | 2/23/2020 | 4/3/2020 | 10.43493 | -84.00412 |
|  |  | 1.2 | 2 | 1 | 6/26/2021 | 6/27/2021 | 10.43493 | -84.00412 |
| *P. umbricola* | gap; early-suc | 1 | 7+ | 30 | 1/26/2020 | 2/25/2020 | 10.43183 | -84.006 |
|  |  | 2 | 3 | 8 | 2/18/2020 | 2/26/2020 | 10.43153 | -84.00594 |

**Table 2.** Parameters of search-phase echolocation call focal data of *C. castanea*, *C. perspicillata*, and *C. sowelli* calculated using RavenPro v. 1.6.2’s spectrogram display and analysis capabilities (512 FFT Hanning window, 95% overlap; see Methods).

| **Species**  **Sample size** |  | **Delta Time (ms)** | **Dur 90% (ms)** | **Low Freq (kHz)** | **High Freq (kHz)** | **Freq 95% (kHz)** | **Peak Freq (kHz)** | **Delta Freq (kHz)** | **BW 90% (kHz)** |
| --- | --- | --- | --- | --- | --- | --- | --- | --- | --- |
| *C. castanea*  N= 255 | Average | 1.18 | 0.615 | 66.3 | 95 | 91.7 | 80.2 | 28.7 | 20.4 |
|  | Standard deviation | 0.295 | 0.286 | 4.94 | 6.34 | 6.2 | 7.87 | 7.78 | 6.35 |
|  | 66% | 0.887 - 1.48 | 0.328 - 0.901 | 61.4 - 71.2 | 88.7 - 101.3 | 85.5 - 97.9 | 72.4 - 88.1 | 20.9 - 36.5 | 14.1- 26.8 |
|  | Min - max | 0.5 - 2.1 | 0.0 - 1.4 | 58.5 - 85.8 | 79.9 - 115.0 | 78.4- 112.0 | 65.9 - 102.0 | 12.0 - 45.6 | 8.79 - 36.6 |
| *C. perspicillata*  N = 28 | Average | 1.06 | 0.468 | 52.2 | 77.6 | 71.1 | 66 | 25.3 | 13.5 |
|  | Standard deviation | 0.211 | 0.261 | 4.61 | 3.12 | 3.08 | 3.41 | 4.68 | 4.03 |
|  | 66% | 0.849 - 1.27 | 0.207 - 0.729 | 47.6 - 56.8 | 74.5 - 80.7 | 68.0 - 74.1 | 62.6 - 69.4 | 20.6 - 30.0 | 9.48 - 17.5 |
|  | min - max | 0.5 - 1.5 | 0 - 1.0 | 45.1 - 59.6 | 71.5 - 84.8 | 63.5 - 75.4 | 57.6 - 75.2 | 16.7 - 34.7 | 7.81 - 23.4 |
| *C. sowelli*  N = 69 | Average | 1.28 | 0.386 | 59.7 | 79.2 | 76.4 | 70.9 | 19.5 | 12 |
|  | Standard deviation | 0.33 | 0.371 | 7.08 | 4.43 | 4.42 | 5.02 | 5.81 | 3.22 |
|  | 66% | 0.952 - 1.61 | 0.015 - 0.756 | 52.6 - 66.8 | 74.8 - 83.6 | 72.0 - 80.8 | 65.9 - 75.9 | 13.7 - 25.3 | 8.78 - 15.2 |
|  | min - max | 0.6 - 2.2 | 0 - 1.4 | 45.0 - 76.8 | 71.0 - 88.6 | 66.7 - 85.7 | 56.4 - 81.3 | 9.50 - 29.8 | 6.59 - 18.3 |

**Table 3.** Number of files identified as likely containing bat calls after filtering by MATLAB signal processing algorithm with more sensitive parameters (Run 1, low pass 100kHz, high pass 20kHz, peak frequency 40kHz) and with more specific parameters (Run 2, low pass 110kHz, high pass 30kHz, peak frequency 50kHz).

| **Species** | **Site** | **Total files** | **Post Run 1** | **% reduction** | **Post Run 2** | **% reduction** | **N files (Run 1 + Run 2)** |
| --- | --- | --- | --- | --- | --- | --- | --- |
| *P. auritum* | 1 | 6120 | 466 | 92.39 |  |  | 466 |
| *P. colonense* | 1 | 1626 | 54 | 96.67 | 346 | 96.68 | 400 |
|  | 2 | 1440 | 270 | 81.25 | 498 | 65.42 | 768 |
|  | 3 | 9090 | 201 | 97.79 | 923 | 89.85 | 1124 |
| *P. cyanophyllum* | 1 | 6307 | 123 | 98.05 | 87 | 98.62 | 210 |
| *P. generalense* | 1 | 6321 | 124 | 98.03 | 171 | 97.29 | 295 |
|  | 2 | 6327 | 126 | 98 | 170 | 97.31 | 296 |
|  | 3 | 6327 | 125 | 98.02 | 172 | 97.28 | 297 |
|  | 4 | 4321 | 87 | 97.99 | 142 | 96.71 | 229 |
|  | 5 | 7099 | 240 | 96.62 | 504 | 92.9 | 744 |
|  | 6 | 6314 | 134 | 97.88 | 222 | 96.48 | 356 |
|  | 7 | 6314 | 479 | 92.41 | 222 | 96.48 | 701 |
|  | 8 | 6221 | 123 | 98.02 | 87 | 98.6 | 210 |
| *P. multiplinervium* | 1 | 9360 | 343 | 96.33 | 931 | 90.05 | 1274 |
| *P. nudifolium* | 1 | 4101 | 90 | 97.81 | 77 | 98.12 | 167 |
| *P. paulowniifolium* | 1 | 10800 | 470 | 95.64 | 1767 | 83.64 | 2237 |
|  | 2 | 10800 | 1987 | 81.6 | 1767 | 83.64 | 3754 |
|  | 3 | 9000 | 164 | 98.18 | 936 | 89.6 | 1100 |
| *P. reticulatum* | 1 | 5040 | 89 | 98.23 | 150 | 97.02 | 239 |
|  | 2 | 2362 | 42 | 98.22 | 35 | 98.52 | 77 |
|  | 3 | 1440 | 270 | 81.25 | 706 | 50.97 | 976 |
|  | 4 | 1440 | 150 | 89.58 | 706 | 50.97 | 856 |
|  | 6 | 1440 | 38 | 97.36 | 706 | 50.97 | 744 |
|  | 7 | 4318 | 466 | 89.21 | 803 | 50.97 | 1269 |
|  | 8 | 2470 | 0 | 100 | 789 | 68.06 | 789 |
|  | 9 | 9000 | 164 | 98.18 | 933 | 89.63 | 1097 |
|  | 10 | 6211 | 4 | 99.93 | 730 | 88.25 | 734 |
| *P. sancti-felicis* | 1 | 1080 | 18 | 98.33 | 33 | 96.94 | 51 |
|  | 2 | 1080 | 22 | 97.96 | 64 | 94.07 | 86 |
|  | 3 | 1080 | 28 | 97.4 | 38 | 96.5 | 66 |
|  | 4 | 359 | 2 | 99.44 | 2 | 99.44 | 4 |
|  | 5 | 1222 | 47 | 96.15 | 262 | 78.56 | 309 |
|  | 7 | 3508 | 72 | 97.95 | 92 | 97.38 | 164 |
|  | 8 | 1649 | 66 | 95.99 | 44 | 97.33 | 110 |
|  | 11 | 4676 | 902 | 80.71 | 737 | 84.24 | 1639 |
| *P.*sp*.* D | 1 | 6711 | 614 | 90.85 | 89 | 85.5 | 703 |
|  | 2 | 4320 | 95 | 97.8 | 80 | 98.15 | 175 |
|  | 3 | 360 | 38 | 89.44 | 126 | 65 | 164 |
| *P. sublineatum* | 1 | 360 | 0 | 97.22 | 10 | 97.22 | 360 |
| *P. umbricola* | 1 | 6256 | 123 | 98.03 | 87 | 98.61 | 210 |
|  | 2 | 2880 | 58 | 97.99 | 25 | 99.13 | 83 |
| Average |  | 4564.63 | 217.41 | 95.12 | 406.73 | 87.56 | 622.76 |
| Standard deviation |  | 3047.37 | 342.43 | 5.37 | 450.89 | 15.27 | 710.39 |

**Table 4.** Number of each call type analyzed from acoustic data across 41 plants and 12 *Piper* species (see Methods).

| **Species** | **Site** | **N files** | **Ins (FM-QCF)** | **Ins (FM-CF)** | **Feeding buzz** | **FM with harmonics** | **FM with harmonics (not Carollia)** | **FM without harmonics** | ***Carollia* calls** |
| --- | --- | --- | --- | --- | --- | --- | --- | --- | --- |
| *P. auritum* | 1 | 466 | 358 | 100 | 8 |  | 2 |  |  |
| *P. colonense* | 1 | 400 | 1 | 4 |  |  |  |  |  |
|  | 2 | 768 | 407 | 11 | 15 |  |  | 6 |  |
|  | 3 | 1124 | 138 | 98 | 8 |  |  | 2 |  |
| *P. cyanophyllum* | 1 | 210 | 54 | 19 | 5 |  | 3 |  |  |
| *P. generalense* | 1 | 295 | 136 | 6 | 11 |  | 2 | 3 | 2 |
|  | 2 | 296 | 104 |  | 5 |  | 3 | 9 |  |
|  | 3 | 297 | 129 | 6 | 8 |  | 3 | 4 |  |
|  | 4 | 229 | 54 |  |  | 1 |  | 1 |  |
|  | 5 | 744 | 214 | 2 | 7 |  | 1 | 1 |  |
|  | 6 | 356 |  |  |  |  |  |  |  |
|  | 7 | 701 | 333 | 21 | 14 |  | 2 |  |  |
|  | 8 | 210 | 51 | 24 | 3 |  |  | 4 |  |
| *P. multiplinervium* | 1 | 1274 | 102 | 15 | 5 |  |  |  |  |
| *P. nudifolium* | 1 | 167 | 4 | 4 |  |  | 2 |  | 1 |
| *P. paulowniifolium* | 1 | 2237 | 307 | 49 | 10 |  | 31 | 2 |  |
|  | 2 | 3754 | 2240 | 116 | 396 | 34 | 17 | 298 |  |
|  | 3 | 1100 | 311 | 48 |  |  | 4 | 3 | 2 |
| *P. reticulatum* | 1 | 239 | 28 | 26 |  |  | 8 | 1 |  |
|  | 2 | 77 | 135 |  | 6 |  |  |  |  |
|  | 3 | 976 | 176 | 2 | 3 |  | 3 |  | 2 |
|  | 4 | 856 | 134 | 42 | 8 |  | 6 | 1 |  |
|  | 6 | 744 | 83 | 38 |  |  | 2 |  |  |
|  | 7 | 1269 | 266 | 11 | 29 |  | 15 | 13 | 3 |
|  | 8 | 789 |  |  |  |  |  |  |  |
|  | 9 | 1097 | 314 | 16 | 24 |  | 7 | 2 |  |
|  | 10 | 734 | 198 | 42 | 10 |  |  | 427 |  |
| *P. sancti-felicis* | 1 | 51 | 8 |  |  |  |  |  |  |
|  | 2 | 86 |  | 7 |  |  |  |  |  |
|  | 3 | 66 | 1 | 4 | 1 |  |  |  |  |
|  | 4 | 4 | 1 |  |  |  |  |  |  |
|  | 5 | 309 | 2 | 8 |  |  |  |  |  |
|  | 7 | 164 | 21 | 7 | 3 |  |  | 5 |  |
|  | 8 | 110 |  |  |  |  |  |  |  |
|  | 11 | 1639 | 240 | 162 | 18 |  | 61 | 10 |  |
| *P.* sp. D | 1 | 703 | 686 | 74 | 11 |  | 2 | 2 |  |
|  | 2 | 175 | 7 | 68 | 1 |  |  |  |  |
|  | 3 | 164 | 37 | 62 |  |  |  |  |  |
| *P. sublineatum* | 1 | 360 | 10 | 2 |  |  |  |  |  |
| *P. umbricola* | 1 | 210 | 16 | 37 |  |  | 1 |  |  |
|  | 2 | 83 | 10 | 27 |  |  |  |  |  |

**Table S5.** Diet data compiled from the literature (Santana et al., 2021; Lopez & Vaughan, 2007; Maynard et al., 2019), where hundreds of fecal samples were collected and the percent of each *Piper* species in the fecal samples of each *Carollia* species (*C. sowelli, C. castanea, C. perspicillata*) were determined. We included the habitat classification for each *Piper* species.

| **Species** | **Habitat**  **Classification** | | ***C. sowelli*** | ***C. castanea*** | ***C. perspicillata*** | **Max. %** | **Average %** |
| --- | --- | --- | --- | --- | --- | --- | --- |
| *P. aduncum* | open | mid-suc | 1.519 | 1.1013 | 0.8658 | 1.519 | 1.162 |
| *P. aequale (cabagranum)* | closed | late-suc | 0 | 0 | 0 | 0 | 0 |
| *P. prismaticum (augustum)* | closed | late-suc | 1.2658 | 0.2203 | 0.4329 | 1.2658 | 0.6397 |
| *P. auritifolium* | closed | late-suc | 0.2532 | 0 | 0 | 0.2532 | 0.0844 |
| *P. auritum* | open | early-suc | 15.19 | 0.8811 | 12.987 | 15.19 | 9.686 |
| *P. colonense* | open | mid-suc | 6.8354 | 3.9648 | 6.061 | 6.8354 | 5.6203 |
| *P. concepcionis* | closed | late-suc | 0.7595 | 1.1013 | 0.8658 | 1.1013 | 0.9089 |
| *P. cyanophyllum (phytolaccifolium)* | closed | mid-suc | 0 | 0.2203 | 0 | 0.2203 | 0.0734 |
| *P. darianense* | closed | mid-suc | 0 | 0 | 0 | 0 | 0 |
| *P. decurrens* | closed | mid-suc | 0 | 0 | 0 | 0 | 0 |
| *P. dryadum* | closed | mid-suc | 0 | 0 | 0 | 0 | 0 |
| *P. garagaranum* | closed | late-suc | 0 | 0 | 0 | 0 | 0 |
| *P. generalense* | closed | mid-suc | 0.2532 | 1.549 | 3.8961 | 3.8961 | 1.8970 |
| *P. holdridgeanum* | closed | late-suc | 0.2532 | 0 | 0 | 0.2532 | 0.0844 |
| *P. evasum (imperiale)* | closed | late-suc | 0 | 0 | 0.4329 | 0.4329 | 0.1443 |
| *P. melanocladum* | closed | late-suc | 0 | 0 | 0 | 0 | 0 |
| *P. multiplinervium* | open | early-suc | 7.089 | 18.282 | 18.615 | 18.615 | 14.662 |
| *P. nudifolium* | closed | mid-suc | 0 | 0.2203 | 0 | 0.2203 | 0.0734 |
| *P. paulowniifolium* | closed | mid-suc | 0.5063 | 2.423 | 0.4329 | 2.423 | 1.1207 |
| *P. peltatum* | open | early-suc | 0.5063 | 0 | 0.8658 | 0.8658 | 0.4574 |
| *P. pentagonum (cenocladum)* | closed | late-suc | 0 | 0 | 0 | 0 | 0 |
| *P. peracuminatum* | closed | mid-suc | 4.3038 | 4.626 | 4.7619 | 4.7619 | 4.5638 |
| *P. reticulatum* | open | mid-suc | 5.3165 | 6.1674 | 3.896 | 6.167 | 5.1267 |
| *P. sancti-felicis* | open | early-suc | 35.443 | 28.634 | 23.377 | 35.443 | 29.151 |
| *P. silvivagum* | closed | early-suc | 0.2532 | 0.8811 | 0.4329 | 0.8811 | 0.5224 |
| *P*. sp. D *(hispidum)* | open | mid-suc | 2.5316 | 3.0837 | 5.1948 | 5.1948 | 3.6034 |
| *P. sublineatum* | closed | mid-suc | 0.5063 | 0 | 0 | 0.5063 | 0.1688 |
| *P. terrabanum* | closed | mid-suc | 0.2532 | 0.4405 | 0 | 0.4405 | 0.2312 |
| *P. tonduzii* | closed | late-suc | 0 | 0 | 0 | 0 | 0 |
| *P. umbricola* | open | early-suc | 7.595 | 11.454 | 6.4935 | 11.454 | 8.5141 |
| *P. urophyllum* | closed | mid-suc | 0 | 0.6608 | 0 | 0.6608 | 0.2203 |
| *P. urostachyum* | closed | late-suc | 0.7595 | 0.4405 | 0.4329 | 0.7595 | 0.5443 |
| *P. xanthostachium* | closed | mid-suc | 0 | 0 | 0 | 0 | 0 |

**Table S6:** *Piper* species from the chemical dataset published in Santana et al. 2021, sorted by the concentration per species for each of the 15 most abundant chemicals among all *Piper* plants in the study.

| **Species** | **N** | **Number of VOCs** | **Total emissions** | **Alpha caryophyllene** | **Germacrene D** | **Humulene** | **Beta pinene** | **Beta ocimene** | **Ethyl ester benzoic acid** |
| --- | --- | --- | --- | --- | --- | --- | --- | --- | --- |
| *P. aduncum* | 4 | 45 | 2149038.8 | 50771.502 | 0 | 29129.335 | 36700.016 | 88865.07 | 0 |
| *P. auritifolium* | 3 | 30 | 54551.65 | 6723.027 | 188.517 | 10891.9 | 0 | 0 | 0 |
| *P. colonense* | 7 | 53 | 336086.12 | 13638.425 | 88.5674 | 14489.94 | 15016.16 | 0 | 0 |
| *P. concepcionis* | 1 | 15 | 1012491.1 | 6691.717 | 122027.89 | 0 | 0 | 67192.26 | 24685.25 |
| *P. darienense* | 2 | 16 | 91849.461 | 1464.618 | 0 | 0 | 0 | 0 | 1196.224 |
| *P. evasum* | 1 | 4 | 26157.17 | 0 | 19647.072 | 0 | 0 | 0 | 0 |
| *P. garagaranum* | 1 | 6 | 358300.82 | 70349.76 | 0 | 64729.416 | 0 | 167698.85 | 0 |
| *P. generalense* | 5 | 30 | 260330.51 | 16212.934 | 139114.65 | 11815.696 | 0 | 2645.9689 | 1815.836 |
| *P. multiplinervium* | 4 | 24 | 278168.25 | 89853.81 | 37333.27 | 1291.501 | 21862.52 | 0 | 13432.78 |
| *P. nudifolium* | 2 | 24 | 103636.34 | 56986.59 | 0 | 5570.728 | 0 | 664.44 | 0 |
| *P. paulowniifolium* | 3 | 34 | 2040145.5 | 331420.80 | 160401.28 | 27031.66 | 252901.83 | 0 | 65289.071 |
| *P. peltatum* | 7 | 30 | 94475.32 | 6743.055 | 0 | 306.84 | 15529.17 | 0 | 3304.495 |
| *P. peracuminatum* | 6 | 54 | 77919.446 | 4753.209 | 9563.819 | 2518.379 | 24410.341 | 7672.922 | 0 |
| *P. prismaticum* | 1 | 7 | 12864.523 | 2560.994 | 4213.658 | 0 | 0 | 2061.808 | 0 |
| *P. reticulatum* | 7 | 38 | 388150.5 | 145054.28 | 153706.94 | 14124.21 | 6289.492 | 11345.25 | 0 |
| *P. sanctifelicis* | 34 | 104 | 332076.55 | 13490.87 | 2353.9581 | 1197.346 | 4414.296 | 3486.585 | 1505.972 |
| *P. silvivagum* | 4 | 38 | 901877.65 | 79779.821 | 256428.71 | 21961.053 | 3498.3841 | 3076.921 | 8479.863 |
| *P.* sp. D | 1 | 14 | 186638.94 | 54078.504 | 44288.098 | 4529.4834 | 27512.777 | 1985.11 | 2460.496 |
| *P. sublineatum* | 2 | 19 | 121389.49 | 2443.3969 | 8659.433 | 0 | 4196.9461 | 0 | 0 |
| *P. umbricola* | 2 | 23 | 579672.39 | 90529.270 | 249183.05 | 12642.580 | 34944.971 | 18569.177 | 35065.723 |
| *P. urostachyum* | 5 | 37 | 817541.97 | 17663.48 | 90049.41 | 1920.305 | 4895.161 | 0 | 5522.61 |

| **Species** | **N** | **Alpha cubebene** | **Alpha phellandrene** | **p-Cymene** | **1-Dodecene** | **Beta elemene** | **3-methyl-2-undecene** | **3-methyl-3-undecene** | **Decanal** | **Beta myrcene** |
| --- | --- | --- | --- | --- | --- | --- | --- | --- | --- | --- |
| *P. aduncum* | 4 | 0 | 90186.92 | 131884.5 | 19022.09 | 19495.38 | 7933.617 | 2992.274 | 6066.076 | 4101.439 |
| *P. auritifolium* | 3 | 534.818 | 0 | 105.462 | 645.632 | 83.855 | 332.6398 | 117.161 | 157.494 | 0 |
| *P. colonense* | 7 | 194.139 | 139964.4 | 17509.65 | 3787.228 | 29271.40 | 390.8159 | 1517.286 | 3262.210 | 8446.168 |
| *P. concepcionis* | 1 | 0 | 7345.35 | 6562.018 | 0 | 0 | 0 | 0 | 0 | 11083.62 |
| *P. darienense* | 2 | 0 | 0 | 0 | 0 | 0 | 0 | 0 | 0 | 0 |
| *P. evasum* | 1 | 0 | 0 | 0 | 0 | 3070.262 | 0 | 0 | 0 | 0 |
| *P. garagaranum* | 1 | 22187.5 | 0 | 0 | 0 | 0 | 0 | 0 | 0 | 0 |
| *P. generalense* | 5 | 1608.184 | 0 | 0 | 0 | 2890.77 | 0 | 0 | 0 | 0 |
| *P. multiplinervium* | 4 | 0 | 14631.05 | 0 | 4892.057 | 3358.014 | 1855.747 | 1852.005 | 1829.407 | 0 |
| *P. nudifolium* | 2 | 0 | 0 | 5068.43 | 5963.092 | 0 | 2282.932 | 2253.293 | 598.257 | 0 |
| *P. paulowniifolium* | 3 | 490904.4 | 18932.38 | 69458.10 | 0 | 0 | 0 | 0 | 0 | 10780.71 |
| *P. peltatum* | 7 | 1716.442 | 302.187 | 8947.197 | 0 | 0 | 0 | 0 | 0 | 112.238 |
| *P. peracuminatum* | 6 | 351.744 | 5114.928 | 1271.054 | 2528.012 | 429.074 | 1569.782 | 1024.091 | 654.015 | 731.686 |
| *P. prismaticum* | 1 | 0 | 0 | 0 | 0 | 0 | 0 | 0 | 0 | 0 |
| *P. reticulatum* | 7 | 4942.997 | 1950.65 | 0 | 4725.67 | 0 | 2353.194 | 1641.333 | 1464.82 | 0 |
| *P. sanctifelicis* | 34 | 29447.1 | 50316.12 | 13395.3 | 11352.69 | 1732.81 | 15062.26 | 10314.81 | 1600.92 | 20.1366 |
| *P. silvivagum* | 4 | 20310.03 | 907.461 | 0 | 0 | 0 | 0 | 0 | 0 | 2283. 9 |
| *P. spD* | 1 | 4152.43 | 0 | 11195. 9 | 0 | 18763.88 | 0 | 0 | 0 | 1089.261 |
| *P. sublineatum* | 2 | 0 | 0 | 0 | 33461.71 | 0 | 11374.12 | 7864.433 | 2411.604 | 0 |
| *P. umbricola* | 2 | 7012.67 | 0 | 0 | 19288.39 | 3150.01 | 7218.636 | 4301.37 | 13287.46 | 6759.253 |
| *P. urostachyum* | 5 | 6799.87 | 409428.2 | 79098.07 | 0 | 0 | 0 | 0 | 0 | 11518.93 |

References

Barclay, RMR. 1999. Bats are Not Birds—a Cautionary Note on Using Echolocation Calls to Identify Bats: a Comment. J Mammal 80(1):290-296.

Collen A. 2012. The evolution of echolocation in bats: a comparative approach. Doctoral thesis, University College London.

Fraser EE, Silvis A, Brigham RM, Czenze ZJ. 2020. Bat Echolocation Research: A handbook for planning and conducting acoustic studies (2nd ed.). Bat Conservation International, USA.

Griffin DR. 1971. The importance of atmospheric attenuation for the echolocation of bats (Chiroptera). Anim Behav 19(1):55-61.

Jakobsen L, Ratcliffe J, Surlykke A. 2013. Convergent acoustic field of view in echolocating bats. Nat 493:93–96.

Krivek G, Schulze B, Poloskei PZ, Frankowski K, Mathgen X, Douwes A, van Schaik J. 2022. Camera traps with white flash are a minimally invasive method for long‐term bat monitoring. Remote Sens Ecol Conserv 8(3):284-296.

Lawrence BD, Simmons JA. 1982. Measurements of atmospheric attenuation at ultrasonic frequencies and the significance for echolocation by bats. J Acoust Soc Am 71(3):585-590.

Leiser-Miller LB, Kaliszewska ZA, Lauterbur ME, Mann B, Riffell JA, Sharlene SE. 2020. A Fruitful Endeavor: Scent Cues and Echolocation Behavior Used by Carollia castanea to Find Fruit. Integr Org Biol 2:obaa007.

Lopez JE, Vaughan C. 2007. Food niche overlap among neotropical frugivorous bats in Costa Rica. Rev Biol Trop 55:301–13.

Maynard LD, Ananda A, Sides MF, Burk H, Whitehead SR. 2019. Dietary resource overlap among three species of frugivorous bat in Costa Rica. J Trop Ecol 35:165–72.

Neuweiler G. 1990. Auditory adaptations for prey capture in echolocating bats. Physiol Rev 70(3):615-41.

Obrist MK. 1995. Flexible bat echolocation: the influence of individual, habitat and conspecifics on sonar signal design. Behav Ecol Sociobiol 36:207–219.

Russo D, Voigt CC. 2016. The use of automated identification of bat echolocation calls in acoustic monitoring: A cautionary note for a sound analysis. Ecol Indic 66:598-602.

Santana SE, Kaliszewska ZA, Leiser-Miller LB, Lauterbur ME, Arbour JH, Dávalos LM, Riffell JA. 2021. Fruit odorants mediate co-specialization in a multispecies plant–animal mutualism. Proc R Soc B, 288:20210312.

Schnitzler H, Kalko EKV. 2001. Echolocation by Insect-Eating Bats: We define four distinct functional groups of bats and find differences in signal structure that correlate with the typical echolocation tasks faced by each group, Biosci 51(7):557-569.

Sollmann R, Mohamed A, Samejima H, Wilting A. 2013. Risky business or simple solution–Relative abundance indices from camera-trapping. Biol Conserv 159:405-412.
